# Sleep and Its Impact on the Success of Weight Loss Interventions in Adults With Excess Weight: A Systematic Review

**DOI:** 10.1111/obr.70199

**Published:** 2026-07-20

**Authors:** Lauren Duan, Jessica Berryhill, Nichole Dai, Gabrielle Sica, Jean‐Philippe Chaput

**Affiliations:** ^1^ School of Epidemiology and Public Health, Faculty of Medicine University of Ottawa Ottawa Ontario Canada; ^2^ Healthy Active Living and Obesity Research Group Children's Hospital of Eastern Ontario Research Institute Ottawa Ontario Canada; ^3^ Department of Pediatrics, Faculty of Medicine University of Ottawa Ottawa Ontario Canada

**Keywords:** bariatric surgery, behavioral weight loss interventions, obesity management, sleep duration, sleep health, sleep satisfaction, weight management

## Abstract

**Objective:**

To evaluate evidence on the impact of sleep health on weight loss in adults with excess weight undergoing behavioral or surgical weight loss interventions.

**Methods:**

We conducted a systematic review of randomized controlled trials and quasi‐experimental studies including adults aged ≥ 18 years with BMI ≥ 25 kg/m^2^. MEDLINE, Embase, and CENTRAL were searched from inception to September 2025. Screening, data extraction, and risk of bias assessment were performed in duplicate. Sleep was examined either as an exposure or as an intervention component. A narrative synthesis was conducted using the RU‐SATED framework and stratified by sleep exposure versus sleep intervention studies.

**Results:**

Thirty studies (13 randomized controlled trials and 17 quasi‐experimental studies; 10,944 participants from 13 countries) were included. Evidence was mixed across sleep dimensions. In behavioral weight loss interventions, sleep manipulation studies suggested that sleep restriction reduced fat loss, whereas observational studies reported inconsistent associations between sleep duration and weight loss. Limited evidence suggested that greater sleep satisfaction and higher sleep efficiency may support weight loss, with approximately half of the studies reporting positive associations. Evidence for sleep regularity, timing, and alertness was sparse and inconsistent because of the small number of studies and methodological heterogeneity.

**Conclusion:**

Sleep satisfaction and sleep efficiency may support weight loss in behavioral interventions, whereas sleep duration shows inconsistent associations and sleep restriction may impair fat loss. Evidence for sleep regularity, timing, and alertness remains limited. High‐quality experimental studies are needed to clarify the causal role of sleep health in weight loss outcomes.

**Abbreviations**
BMI: body mass index
ESS: Epworth Sleepiness Scale
PICOS: Populations, Interventions, Comparisons, Outcomes, and Study Design Framework
PRISMA: Preferred Reporting Items for Systematic Reviews and Meta‐Analyses
PSQI: Pittsburgh Sleep Quality Index
RCT: randomized controlled trial
RoB‐2: Cochrane Risk of Bias 2 Tool
ROBINS‐I: Cochrane Risk of Bias in Non‐Randomized Studies of Interventions Tool

## 1 Introduction

Obesity continues to be a significant health challenge with a large societal burden around the world [1, 2]. Globally, approximately two billion adults have overweight or obesity, defined as having a body mass index (BMI) greater than 25 kg/m^2^ [2, 3]. Excess weight refers to both overweight and obesity BMI categories and is proven to be an important predictor of serious chronic health conditions and mortality [4]. Adverse health outcomes associated with excess weight include cardiovascular disease, type 2 diabetes mellitus, nonalcoholic fatty liver disease, musculoskeletal disorders, and mental health disorders that impact the quality of life of patients [4, 5]. Excess weight is thought to be influenced by an interaction between genetic, environmental, and lifestyle factors; in particular, sleep has been identified as a potential risk factor for weight gain [6]. For instance, insufficient sleep duration has been shown to be an independent risk factor for incident obesity and weight gain in adult populations [7, 8].

Sleep health is an all‐encompassing term that includes dimensions such as sleep duration, satisfaction, efficiency, regularity, timing, and alertness; it is not simply the absence of clinical sleep disorders such as insomnia or obstructive sleep apnea [9]. Considering the effects of sleep on various physiological mechanisms including metabolism and hunger, many dimensions of sleep health have been associated with weight gain. For example, sleep restriction and poor sleep quality have been shown to increase the risk of obesity and weight gain through higher energy consumption and alterations in food choices [10, 11].

For adults with excess weight, behavioral weight loss interventions are a common method to manage obesity and are usually composed of two main strategies: reduced energy intake and increased physical activity. Pharmacological and surgical interventions such as weight loss drugs and bariatric surgery are also utilized for weight management. Despite strong scientific evidence supporting the relationship between sleep health and weight, sleep health improvement is often not integrated into weight loss programs. The relationship between sleep health and weight loss in adults with excess weight undergoing a weight loss intervention is also not well understood. In recent years, there has been an increasing number of experimental studies investigating this phenomenon; however, there are currently no systematic reviews on this topic.

This systematic review aimed to evaluate the available evidence regarding the impact of sleep health (e.g., duration, satisfaction, efficiency, and timing) on weight loss outcomes in adults aged 18 years or older with excess weight undergoing a weight loss intervention. Findings from this review will help inform the development of more effective obesity management interventions for adults with excess weight. In addition, findings from this review will help inform public health guidelines and programming, raising awareness of the importance of sleep health in weight management. Finally, this systematic review will also help evaluate the current state of scientific evidence regarding sleep health and weight loss and identify research gaps.

## 2 Methods

### 2.1 Protocol and Registration

The present systematic review was registered a priori with Open Science Framework (available from https://osf.io/mvd5x/) and was conducted in accordance with the Preferred Reporting Items for Systematic Reviews and Meta‐Analyses (PRISMA) 2020 statement [12].

### 2.2 Eligibility Criteria

The Populations, Interventions, Comparisons, Outcomes, and Study Design (PICOS) framework [13] was used to identify key concepts in the research question a priori and to guide the search strategy. Studies were selected for review based on the criteria detailed below.

#### 2.2.1 Population

We included studies examining adults ≥ 18 years of age with excess weight (overweight or obesity) who are undergoing a weight loss intervention. For this review, a BMI of ≥ 25 kg/m^2^ and ≥ 30 kg/m^2^ were considered overweight and obesity, respectively. We excluded studies with pregnant and hospitalized populations. A weight loss intervention was defined as any structured intervention aimed at weight/adiposity loss using physical activity, dietary, pharmacological, or surgical means. This definition included interventions based solely on structured physical activity because physical activity is an established component of obesity management and may result in weight loss.

#### 2.2.2 Exposure/Intervention

Studies must include at least one dimension of sleep health (e.g., duration, satisfaction, regularity, timing, and alertness) as an exposure or intervention. For intervention studies, eligibility required that the intervention directly evaluated the role of a sleep health dimension in relation to weight loss outcomes. Studies in which sleep promotion was incorporated as one component of a broader multicomponent behavioral intervention, without evaluating the specific contribution of sleep, were not eligible. In RCTs, sleep health may be a component of the overall weight loss intervention (e.g., assigned sleep duration group). Otherwise, sleep health may be measured as an exposure at baseline and/or throughout the weight loss intervention. Sleep health may be measured using either objective (e.g., polysomnography and actigraphy) or subjective (e.g., self‐report questionnaires) methods.

#### 2.2.3 Comparator

A comparator was not necessary, but studies comparing various levels of sleep between intervention groups remained eligible for inclusion. In most studies, sleep is not part of the weight loss intervention but is assessed as an exposure, meaning that it was measured at baseline and throughout the study. In these studies, the association between baseline sleep health and subsequent weight loss from the weight loss intervention was assessed. However, there may be studies where sleep is part of the weight loss intervention, for example, in studies that restricted sleep duration. In this case, comparator groups may exist to compare the success of the weight loss intervention in groups assigned 8 h versus 5 h of sleep per night, for example. Both types of studies were included in our review.

#### 2.2.4 Outcomes

We included studies that reported at least one objective (e.g., using digital scales and dual‐energy X‐ray absorptiometry) or subjective (e.g., self‐report) measure of weight/adiposity loss. Measures of weight and adiposity loss include but are not limited to changes in body weight, BMI, fat percentage, fat mass, and waist circumference.

#### 2.2.5 Study Design

Only randomized controlled trials (RCTs) and quasi‐experimental studies (e.g., pre–post studies) were included in our review.

#### 2.2.6 Study Language, Publication Status, and Timeframe

We only included studies in English and French based on the language proficiencies of the review team. Studies must be published or in press peer‐reviewed articles. No gray literature (e.g., conference abstracts and book chapters) was included due to the lack of peer review. There were no sample size limitations. All studies regardless of publication date and setting were included.

### 2.3 Information Sources

We conducted searches for eligible studies within the following databases:

- MEDLINE (1946 to February 20, 2024)
- Embase (1947 to February 20, 2024)
- CENTRAL (up to February 20, 2024)

We used the OVID interface for MEDLINE and Embase and the Cochrane Library for CENTRAL. Due to the composite nature of CENTRAL, there is no official inception date.

An updated search was conducted on September 21, 2025, using the same methodology. No additional date limits were imposed, and all citations were imported to Covidence for de‐duplication and screening.

### 2.4 Search Strategy

Five target articles were used to search for records within databases. Search terms were developed by extracting keywords from the titles and abstracts of those studies. Only quantitative studies were sought. There were search limits on language (English and French) and study design (no conference abstracts, case reports, editorials, or letters). In collaboration with a research librarian, a draft search strategy was developed in MEDLINE and subsequently translated to other databases (see Supporting Information). To validate the search strategy, we verified that all five target articles were identified. Peer review using the PRESS checklist was not conducted [14].

### 2.5 Selection Process

Results of final searches in each database were exported to Covidence, where all reviewers were granted access to collaboratively screen articles. Duplicates were removed by a built‐in algorithm in Covidence. Additionally, final search results were uploaded to Zotero to allow for collaborative citation management during the review.

Using Covidence and the eligibility criteria, a two‐stage screening process was conducted [15]. In Level 1 screening, 30 articles were piloted in Covidence to ensure consistency across reviewers. Subsequently, all titles and abstracts were screened by two independent reviewers (two of the following authors: L.D., J.B., N.D., and G.S.). A study was excluded in Level 1 screening when both reviewers classified the article as “No.” Full texts of all potentially eligible studies were retrieved for Level 2 screening. Five articles were piloted to ensure consistency, and all full‐text articles were screened by two independent reviewers (two of the following authors: L.D., J.B., N.D., and G.S.). Discordant results were resolved via discussion with all contributors and by a third reviewer when necessary. All reasons for full‐text article exclusion were documented. The reviewers were not blinded to the name of the journal, authors, or institutions involved in each publication.

### 2.6 Data Collection Process

Microsoft Excel was used for data extraction and data storage. A data extraction matrix was used to facilitate uniform data collection methods. The data extraction form was piloted using two selected articles to ensure uniformity across the interpretation of data items to be collected. Subsequently, two independent reviewers (two of the following authors: L.D., J.B., and G.S.) completed data extraction for each eligible study. Discrepancies were resolved via discussion and by a third independent reviewer if necessary.

### 2.7 Data Items (Outcomes)

The outcome of interest is weight loss; measures of weight loss include but are not limited to changes in body weight, BMI, fat mass, and waist circumference. Objective measurements of weight loss (e.g., digital scales) were prioritized over subjective measures (e.g., self‐report). All outcome measurement timepoints were extracted, but measurements at the end of the study were prioritized in analysis.

### 2.8 Data Items (Other Variables)

We extracted information about the first author, country, funding sources, and the year and source of publication. For all studies, we extracted the study design, sample size, type of weight loss intervention, duration of intervention, duration of follow‐up, and reported effect size. Participant baseline characteristics were also extracted. We extracted all reported measurements of sleep health dimensions and key findings relevant to the weight/adiposity loss outcome.

### 2.9 Risk of Bias

For RCTs, risk of bias assessment was conducted using the Cochrane Risk of Bias 2 tool (RoB‐2). RoB‐2 addresses the following five domains: Domain 1 (bias arising from the randomization process), Domain 2 (bias due to deviations from intended interventions), Domain 3 (bias due to missing outcome data), Domain 4 (bias in measurement of the outcome), and Domain 5 (bias in selection of the reported result).

For nonrandomized studies, risk of bias assessment was conducted using the Cochrane Risk Of Bias In Non‐randomized Studies of Interventions tool (ROBINS‐I). ROBINS‐I addresses the following seven domains: Domain 1 (bias due to confounding), Domain 2 (bias in selection of participants into the study), Domain 3 (bias in classification of interventions), Domain 4 (bias due to deviations in intended interventions), Domain 5 (bias due to missing data), Domain 6 (bias in measurement of outcomes), and Domain 7 (bias in selection of the reported results).

Prior to risk of bias assessment, each of the RoB‐2 and ROBINS‐I tools was piloted using three included studies. Risk of bias was assessed by two independent reviewers (L.D. and N.D.). Conflicts were resolved via discussion between the two independent reviewers. Following the RoB‐2 algorithm, risk of bias judgments (low, some concerns, and high) were determined for each domain and for the overall study. For ROBINS‐I, risk of bias judgments included low, moderate, serious, and critical.

### 2.10 Reporting Bias Assessment

Assessment of risk of bias due to selective nonreporting was covered in Domain 5 of the RoB‐2 tool. This domain examines whether data analysis was conducted according to a prespecified plan (e.g., protocol and statistical analysis plan). It also looks at whether certain outcome measurements (e.g., timepoints and instruments) and statistical tests were performed but not reported in the published article, potentially due to nonsignificant results.

If a meta‐analysis with at least 10 studies was conducted, we planned to assess publication bias using an examination of funnel plots produced by Egger's and Begg's tests. However, no meta‐analysis was completed, and therefore, there was no formal assessment of publication bias.

### 2.11 Certainty of Evidence

The overall certainty of evidence was not assessed using the GRADE (Grading of Recommendations, Assessment, Development and Evaluation) approach, as this review aimed to synthesize and critically appraise the available evidence rather than develop clinical practice guidelines. Instead, we followed rigorous risk of bias assessments tailored to the study designs included: the Cochrane RoB‐2 tool for RCTs and the ROBINS‐I tool for nonrandomized studies. These instruments provide a detailed evaluation of methodological quality that directly informs the interpretation of study findings.

### 2.12 Synthesis Methods

Study characteristics including study design, sleep health dimensions (e.g., sleep duration and sleep satisfaction), type of weight loss intervention (e.g., behavioral and surgical), and outcomes (e.g., weight loss and adiposity loss) were tabulated to determine eligibility for synthesis. Only studies with the same study design and sleep health dimension were grouped together for synthesis.

A meta‐analysis was not appropriate due to clinical and methodological heterogeneity, particularly due to differences in the types of weight loss interventions, length of the interventions, methods of sleep measurement, and statistical tests conducted. Therefore, a narrative synthesis was conducted. To align our review with the existing sleep literature, we utilized the RU‐SATED framework definition of sleep health, which encompasses the following six dimensions: sleep duration, sleep satisfaction, sleep efficiency, sleep regularity, sleep timing, and alertness [9]. In Section 3, sleep health dimensions were presented in descending order based on the number of studies included.

## 3 Results

### 3.1 Study Characteristics

**FIGURE 1:**
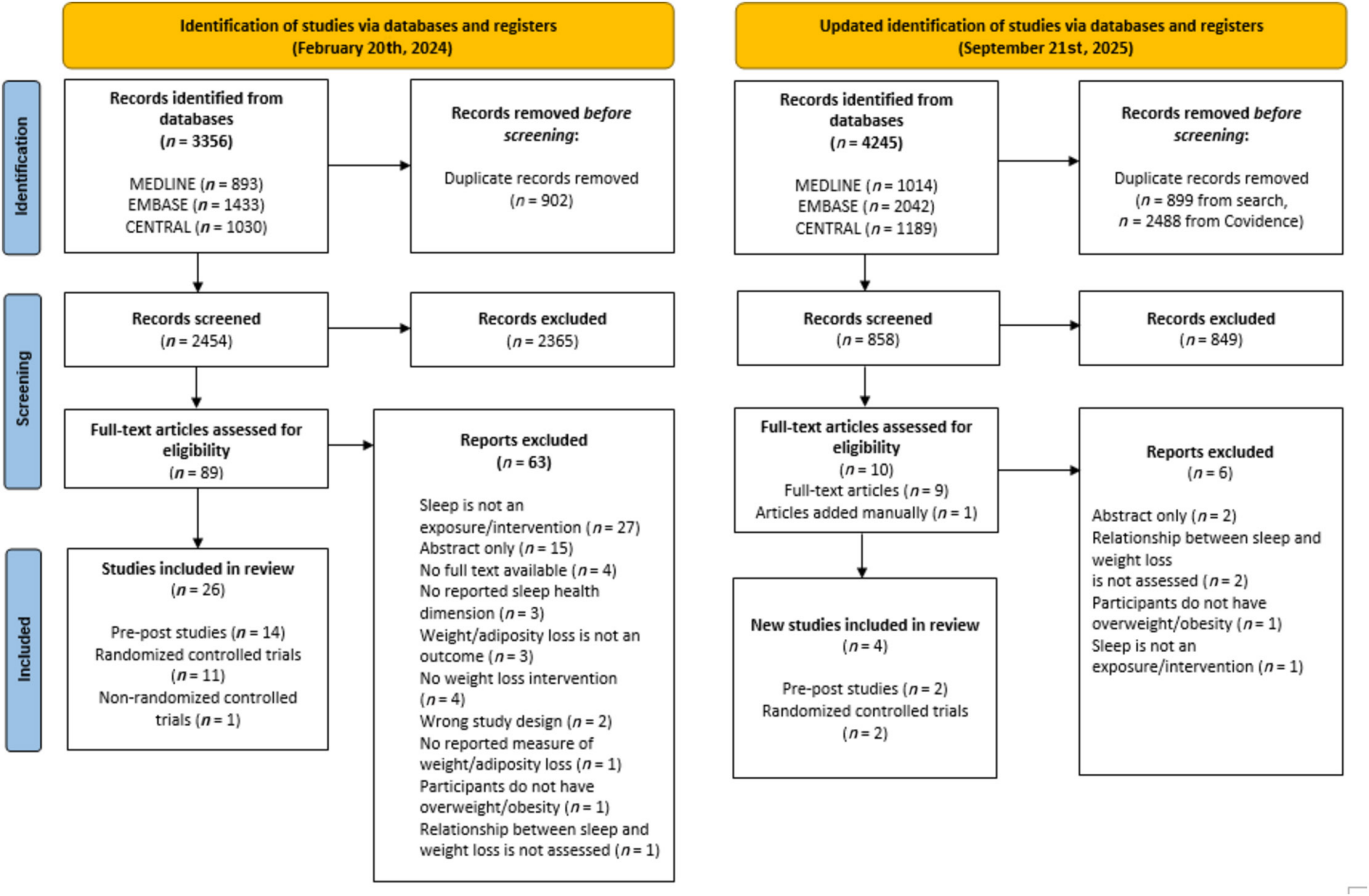
PRISMA 2020 flow diagram on the identification, screening, and inclusion of studies.

A total of 30 studies were included in this review (Figure 1). A total of 13 RCTs investigated the association between sleep duration, sleep satisfaction, sleep efficiency, sleep regularity, sleep timing, and/or alertness and weight loss in adults undergoing weight loss interventions [16, 17, 18, 19, 20, 21, 22, 23, 24, 25, 26, 27, 28]. There were 17 quasi‐experimental studies included, involving 16 pre–post studies [6, 29, 30, 31, 32, 33, 34, 35, 36, 37, 38, 39, 40, 41, 42, 43] and one non‐RCT [44]. All studies were published between 2007 and 2025 with sample sizes ranging from *n* = 10 to *n* = 2990, for a total of *n* = 10,944 participants across all 30 studies (Table 1). Of all studies, three studies comprised only female participants [20, 26, 41]. Twelve studies were conducted in the United States [6, 17, 18, 20, 22, 24, 25, 26, 30, 36, 37, 43], 11 studies were conducted in Europe (Spain, Denmark, Finland, England, Sweden, and the Netherlands) [16, 19, 21, 23, 28, 29, 31, 32, 34, 38, 44], three studies were conducted in Asia (China, Japan, Malaysia) [33, 35, 41], two studies were conducted in Canada [39, 40], one study was conducted in Brazil [42], and one study was conducted in Australia [27].

**TABLE 1:** Baseline characteristics of included studies (*n* = 30).

Author (year)	Study design	Country	Sample size (*n*)	Mean age (years)	Length of follow‐up (weeks)
Altree et al. (2022) [27]	RCT	Australia	80	49.4	52
Bogh et al. (2023) [16]	RCT	Denmark	195	45 in SS 43 in NS	52
Braem et al. (2025) [28]	RCT	Netherlands	34	48.5^a^	26
Fritz et al. (2011) [23]	RCT	Sweden	212	61	12
Li et al. (2022) [24]	RCT	United States	810	51	104
Littman et al. (2007) [17]	RCT	United States	173	61	52
Nedeltcheva et al. (2010) [18]	RCT	United States	10	41	2
O'Brien et al. (2012) [26]	RCT	United States	316	53	24
Papandreou et al. (2019) [19]	RCT	Spain	1986	65	52
Rosas et al. (2020) [25]	RCT	United States	409	51	52
Thomson et al. (2012) [20]	RCT	United States	245	46	104
Tuomilehto et al. (2009) [21]	RCT	Finland	522	55	208
Wang et al. (2018) [22]	RCT	United States	36	45 in CR + SR 45 in CR	8
Marshall et al. (2010) [44]	Nonrandomized controlled trial	Sweden	2990	37–60^b^	520
Barnadas‐Sole et al. (2021) [31]	Pre–post	Spain	252	47.7	52
Carvalho et al. (2025) [42]	Pre–post	Brazil	122	33.0^a^	52
Chaput & Tremblay (2012) [40]	Pre–post	Canada	123	41	24
Creasy et al. (2022) [37]	Pre–post	United States	156	39.7	72
Elder et al. (2012) [6]	Pre–post	United States	472	55	26
Filiatrault et al. (2014) [39]	Pre–post	Canada	150	38.8	16
Garaulet et al. (2013) [38]	Pre–post	Spain	420	42	20
Hansen et al. (2019) [32]	Pre–post	Denmark, Spain, and England	286	46	8
Kline et al. (2021) [36]	Pre–post	United States	125	50.3	52
Mazri et al. (2023) [33]	Pre–post	Malaysia	91	39.6	12
Nielsen et al. (2021) [34]	Pre–post	Denmark	41	39.6	72
Sawamoto et al. (2014) [41]	Pre–post	Japan	90	47.9	28
Swanson et al. (2025) [43]	Pre–post	United States	449	49.9	16
Stromberg et al. (2020) [30]	Pre–post	United States	35	41.2	12
Verhoef et al. (2013) [29]	Pre–post	Netherlands	98	20–50^b^	52
Wen et al. (2023) [35]	Pre–post	China	16	38.1 in SD 34 in NSD	12

Abbreviations: CR, caloric restriction; CR+SR, caloric restriction and sleep restriction; NS, normal sleep; NSD, non‐sleep deprived; RCT, randomized controlled trial; SD, sleep deprived; SS, short sleep.

^a^ Median age is presented due to no reported mean age.

^b^ Age range is presented due to no reported mean age.

Information on the types of weight loss interventions, sleep health dimensions, and key findings of each included study can be found in Table 2. There was variability in the type of weight loss intervention across studies, with the majority including caloric restriction and physical activity. Most behavioral interventions combined dietary modification and physical activity, whereas physical activity‐only interventions were employed by Fritz et al. [23] and Littman et al. [17]. Five studies focused on surgical interventions, including gastric bypass and laparoscopic sleeve gastrectomy [30, 31, 34, 42, 44]. Two of 25 studies with behavioral weight loss interventions included a sleep restriction component [18, 22]. The length of weight loss interventions ranged from 14 days to 4 years. Twelve studies used objective measures of one of the six sleep health dimensions [16, 18, 19, 22, 27, 28, 32, 34, 35, 36, 37, 41], such as actigraphy and polysomnography, whereas the remaining 18 studies [6, 17, 20, 21, 23, 24, 25, 26, 29, 30, 31, 33, 37, 38, 39, 42, 43, 44] relied solely on subjective measures of sleep health, including the Pittsburgh Sleep Quality Index (PSQI) [45, 46], sleep logs, and the Epworth Sleepiness Scale (ESS) [47]. All included studies reported on weight loss with the exception of Chaput and Tremblay [40], who reported exclusively on adiposity loss. In total, 11 studies reported on adiposity loss and mainly used dual‐energy X‐ray absorptiometry for measurement [16, 18, 22, 29, 32, 34, 35, 36, 39, 40, 48]. All studies with weight loss outcomes used an objective measure of weight loss, usually requiring participants to have their weight assessed by a calibrated digital scale during study visits.

**TABLE 2:** Sleep health indicators, interventions, and key findings of included studies sorted by study design (*n* = 30).

Author (year)	Study type	Sleep health indicators	Sleep measurement methods	Intervention	Key findings
Altree et al. (2022) [27]	RCT	Sleep duration Sleep onset latency Sleep efficiency Wake after sleep onset Rise‐time latency Daytime sleepiness Sleep timing	Sleep duration, sleep onset latency, sleep efficiency, wake after sleep onset, and rise time latency were all measured using actigraphy, supplemented by sleep diaries. Daytime sleepiness was measured by the Epworth Sleepiness Scale.	Intervention: Behavioral weight loss intervention including 8 weeks of a very low energy diet (VLED) of 450–800 kcal per day, with 4 weeks of re‐introduction to normal food. After an 8‐week period, one group was instructed to follow a low glycemic index high‐protein diet, whereas the other group was instructed to follow the Australian Guide to Healthy Eating (AGHE) diet. Both diets included a 500‐kcal deficit.	Later bedtime at baseline was associated with higher weight loss at 2 months (rho = −0.45, *p* < 0.001). Change in rise time of a mean of −12.6 ± −45.3 min between baseline and 2 months was associated with higher weight loss at 2 months (rho = 0.36, *p* = 0.03). WASO at 2 months was correlated with weight loss maintenance between 2 and 12 months (rho = −0.39, *p* = 0.02). Correlations related to sleep duration, sleep onset latency, sleep efficiency, rise time latency, and daytime sleepiness were all nonsignificant.
Bogh et al. (2023) [16]	RCT	Sleep duration Sleep quality Sleep efficiency Sleep disturbances	Sleep duration was measured using actigraphy. Sleep quality, sleep efficiency, and sleep disturbances were assessed using the PSQI.	All participants underwent caloric restriction with a limit of 800 kcal/day for 8 weeks before randomization, where only those who lost > 5% of their weight were randomized. Using a 2 × 2 factorial design, participants were either randomized to exercise vs. no exercise, and liraglutide of 3.0 mg/day or placebo for 1 year. The exercise groups were assigned two supervised 45‐min sessions of interval‐based cardio and two 30‐min individual sessions of aerobic exercise every week.	Following the 8‐week diet, for every unit increase in PSQI global score (i.e., poorer sleep quality), participants lost 0.19 kg (*p* = 0.022) less weight. Sleep duration, sleep efficiency, and sleep disturbances were not significantly associated with weight or adiposity loss after the 8‐week diet. Longer sleep duration was associated with greater reductions in body fat percentage (*β* = −0.80, 95% CI = −1.50, −0.10, *p* = 0.026) following the 1‐year intervention.
Braem et al. (2025) [28]	RCT	Sleep duration Sleep efficiency	FitBit Charge 4 (actigraphy)	Intervention: Behavioral weight loss intervention, consisting of personalized dietary advice from professionals and a physical activity program of 30 min/day, 5×/week. The personalized component consisted of personalized advice, self‐monitoring, 360° diagnosis, digital support, feedback based on laboratory results, and blended care. Control: Same as the intervention group, without the personalized component	In the personalized intervention group, longer sleep duration throughout the intervention was correlated with greater weight loss (Spearman's rho = 0.40, *p* < 0.04). There were no significant correlations between sleep efficiency and weight loss.
Fritz et al. (2011) [23]	RCT	Sleep quality	Swedish Health‐Related Quality of Life Questionnaire (SWED‐QUAL)	Intervention: 4‐month behavioral weight loss intervention where participants were instructed to do Nordic walking for 5 h per week. Control: Participants were instructed to maintain normal physical activity levels.	There was no significant correlation between change in sleep quality and change in BMI throughout the weight loss intervention.
Li et al. (2022) [24]	RCT	Sleep disturbance	Standardized symptoms questionnaire	2 × 2 factorial design using calorie‐restricted diets for 2 years. Two diets were high‐fat (40%), and two diets were low‐fat (20%). Two diets were high in protein (25%), and two diets were average in protein (15%). All diets were a 750‐kcal deficit from each participant's baseline resting energy expenditure.	Higher sleep disturbance from baseline to 6 months significantly predicted less weight loss (*p* < 0.001) and a reduction in waist circumference (*p* = 0.002). Repeated measurements of sleep disturbance from baseline to 2 years were negatively associated with overall weight loss rate (weight changes per 6 months) (*p* < 0.001) and changes in waist circumference (*p* < 0.001). Higher sleep disturbance was also associated with lower reductions in total fat (*p* = 0.02), total fat mass percentage (*p* = 0.04), and trunk fat percentage (*p* = 0.05) from baseline to 6 months.
Littman et al. (2007) [17]	RCT	Sleep duration Sleep quality Sleep onset latency Daytime sleepiness	Women's Health Initiative Insomnia Rating Scale Questionnaire	Intervention: 1‐year, behavioral weight loss intervention involving 45 min of moderate‐intensity aerobic exercise, 5 times per week. Control: 45‐min stretching session 1× per week for 1 year, with no changes to normal exercise routines	Exercisers had the most weight loss compared with stretchers when they reported less than 6 h of sleep per night (exercisers vs. stretchers: −2.8 kg, 95% CI: −5.0, −0.6) or poor sleep quality (−3.6, 95% CI: −7.2, 0.1) between baseline and follow‐up. There were no other important exercise‐induced weight loss outcomes related to sleep onset latency or daytime sleepiness.
Nedeltcheva et al. (2010) [18]	RCT	Sleep duration	Polysomnography	Intervention: 2‐week behavioral weight loss intervention with sleep restricted to 5.5 h/night Control: Sleep restricted to 8.5 h/night As a cross‐over trial, participants were randomized to begin with either the 5.5 or 8.5 h per night intervention, followed by the remaining intervention after a 3‐month washout period.	Shorter sleep decreased the proportion of weight lost as fat by 56% (8.5 h: 1.4 kg, 5.5 h: 0.6 kg, *p* = 0.043). Shorter sleep increased loss of fat‐free body mass by 60% (8.5 h: 1.5 kg, 5.5 h: 2.4 kg, *β* = 1.0, 95% CI: 0.4–1.5, *p* = 0.002). Sleep restriction resulted in a significant loss of fat (*β* = − 0.7, 95% CI = −1.4 to −0.03, *p* = 0.043), and percentage weight loss as fat (*β* = −31, 95% CI: −49 to −12, *p* = 0.004).
O'Brien et al. (2012) [26]	RCT	Sleep duration Sleep efficiency Sleep onset latency	PSQI	Intervention: 6‐month behavioral weight loss intervention with hour‐long weekly sessions to set caloric restriction goals between 1200–1500 kcal/day, ≤ 30% of calories from fat, and physical activity goals up to 200 min/per week. Control: Four 1‐h‐long education sessions on weight loss, diet, and exercise	Time in bed (*β* = 0.01) and total sleep duration (*β* = 0.05) did not significantly predict weight loss following the intervention. Less difficulty falling asleep within 30 min at baseline significantly predicted higher subsequent weight loss (*r* = 0.17; *p* < 0.05). No significant association was found between baseline sleep efficiency and weight loss.
Papandreou et al. (2019) [19]	RCT	Sleep duration Sleep efficiency Sleep variability Sleep timing	Actigraphy	Intervention: 12‐month behavioral weight loss intervention consisting of a calorie‐restricted Mediterranean diet and promotion of physical activity and healthy behaviors Control: Mediterranean diet without caloric restriction and no promotion of physical activity	Participants in the third tertile of sleep variability showed a smaller decrease in weight (*p* = 0.020) and BMI at 12 months (*p* = 0.016) compared with participants in the first tertile. Shorter sleep duration was associated with lower waist circumference reduction (*β* = −0.03, 95% CI: −0.5 to—0.04, *p* = 0.024). Sleep efficiency and sleep timing were not significantly associated with changes in weight measures.
Rosas et al. (2020) [25]	RCT	Sleep disturbance	Patient‐Reported Outcomes Measurement Information System (PROMIS) sleep disturbance and sleep‐related impairment scales	Intervention: 12‐month behavioral weight loss intervention with 150 min of moderate‐intensity exercise per week. Participants received nine sessions with a trained health coach, as well as psychotherapy sessions with antidepressant prescription as needed. Control: Routine medical care	Improvements in sleep disturbance were associated with lower BMI at 12 months (*β* = −0.02, 95% CI: −0.04 to 0.00, *p* = 0.03).
Thomson et al. (2012) [20]	RCT	Sleep quality Sleep latency Sleep duration Sleep efficiency Sleep disturbances Daytime sleepiness	PSQI	Intervention: 2‐year behavioral weight loss intervention including a low‐fat and low‐energy diet (1200–2000 kcal/day) and weight loss counseling sessions. Participants were also instructed to partake in 30 min of physical activity five times per week. Control: Participants received weight loss counseling sessions at baseline and 6 months.	Participants with higher subjective sleep quality had a 33% increased likelihood of weight loss success (RR = 0.67, 95% CI: 0.52–0.86). Sleeping > 7 h per night compared with ≤ 7 h was associated with a lower likelihood of successful weight loss at ≥ 10% of baseline weight (RR = 0.70, 95% CI: 0.52–0.86). Poor sleep efficiency (≤ 85%) was associated with a 38% lower likelihood of successful weight loss maintenance at 18 months (RR = 0.62, 95% CI: 0.42–0.92).
Tuomilehto et al. (2009) [21]	RCT	Sleep duration	Sleep logs	Intervention: Behavioral weight loss intervention including personalized counseling with the goal of ≥ 5% weight reduction, ≤ 30% caloric intake from fat, ≤ 10% of caloric intake from saturated fat, fiber intake ≥ 15 g/1000 kcal, and 30 min of moderate‐intensity physical activity daily; the median length of the intervention was 4 years. Control: Participants were given general advice regarding diet and physical activity.	Baseline sleep duration did not significantly predict weight change over a 3‐year period.
Wang et al. (2018) [22]	RCT	Sleep duration	Actigraphy and sleep logs	Intervention: 8‐week behavioral weight loss intervention including caloric restriction of 95% of resting metabolic rate and sleep restriction by reducing time in bed by 90 min per night, 5 days a week. Control: Caloric restriction of 95% of resting metabolic rate only	No statistically significant differences between the sleep restriction and control groups in terms of weight loss. No statistically significant differences between intervention groups for most adiposity loss indicators (fat mass, percentage body fat) except for percentage of total weight loss as fat (*p* = 0.016). In the sleep restriction group, the mean ± SD total weight loss as fat was 16.9% ± 104.5%. The control group reported 80.7% ± 43.3% total weight loss as fat.
Marshall et al. (2010) [44]	Nonrandomized controlled trial	Sleep duration	Self‐reported	Intervention: Surgical weight loss intervention where participants either received gastric bypass, gastric banding, or vertical‐banded gastroplasty. Control: Nonsurgical obesity treatment; this was nonstandardized and varied depending on the healthcare unit. Participants were followed from presurgery to 10 years postsurgery.	Baseline sleep duration did not predict weight loss at any timepoint in either group. Change in sleep duration, assessed both continuously and categorically, was not associated with changes in weight loss in either group.
Barnadas‐Sole et al. (2021) [31]	Pre–post	Sleep duration Sleep timing	Self‐reported	Intervention: Laparoscopic sleeve gastrectomy. Participants were followed from presurgery to 12 months postsurgery.	No association was found between sleep duration and 12‐month percentage excess weight loss. There was a linear association between 1‐h increments of bedtime and 2.23% (95% CI: −3.37 to −0.70, *p* = 0.005) lower excess weight loss. After 12 months, late sleepers (bedtime ≥ 24:00 h) lost 5.64% (95% CI: −10.11 to −1.17, *p* = 0.014) less excess weight compared with early sleepers (bedtime < 24:00 h).
Carvalho et al. (2025) [42]	Pre–post	Sleep quality Daytime sleepiness Social jetlag	Sleep quality was measured using the PSQI. Daytime sleepiness was measured using the Epworth Sleepiness Scale. Social jet lag was measured using self‐reported sleep timing.	Intervention: Surgical weight loss intervention involving Roux‐en‐Y gastric bypass or vertical gastrectomy. Participants were followed from presurgery to 1 year postsurgery.	PSQI score was negatively associated with total weight loss (kg) (3 months: *β* = −0.22; *p* = 0.001, 6 months: *β* = −0.33; *p* < 0.001, 1 year: *β* = −0.33; *p* = 0.002), percentage weight loss (3 months: *β* = −0.24, *p* = 0.005; 6 months: *β* = −0.37, *p* < 0.001; 1 year: *β* = −0.37, *p* = 0.004), and reduced BMI (3 months: *β* = −0.26, *p* = 0.001; 6 months: *β* = −0.34, *p* < 0.001; 1 year: *β* = −0.42, *p* = 0.001). There was also a negative association between daytime sleepiness with weight loss (*β* = −0.28, *p* = 0.009), percentage weight loss (*β* = −0.37, *p* = 0.002), and reduced BMI (*β* = −0.35, *p* = 0.002) at 1 year postsurgery. Social jetlag was negatively associated with total weight loss (kg) at 6 months (*β* = −0.14, *p* = 0.03) and 1 year postsurgery (*β* = −0.24, *p* = 0.03). Social jetlag was also negatively associated with percentage weight loss (6 months: *β* = −0.21, *p* = 0.02; 1 year: *β* = −0.29, *p* = 0.03) and reduced BMI (6 months: *β* = −0.18, *p* = 0.02; 1 year: *β* = −0.25, *p* = 0.03).
Chaput & Tremblay (2012) [40]	Pre–post	Sleep duration Sleep quality	PSQI	The control groups of three behavioral weight loss studies were pooled together in this analysis. All groups underwent a weight loss intervention involving a 600–700 kcal per day reduction in caloric intake. Intervention lengths ranged from 15 weeks to 6 months.	Longer sleep duration was associated with increased loss of body fat (*β* = 0.72, *p* < 0.05). Higher PSQI scores (lower sleep quality) were associated with lower fat mass loss (*β* = −0.19, *p* < 0.05) and percentage body fat loss (*β* = −0.21, *p* < 0.01).
Creasy et al. (2022) [37]	Pre–post	Sleep duration Sleep timing Sleep regularity Sleep efficiency Wake after sleep onset Sleep onset latency Sleep awakenings	SenseWear Mini Armband (actigraphy)	Intervention: 18‐month behavioral weight loss intervention with caloric restriction (1200–1800 kcal/day) and increased physical exercise (300 min/week).	Higher sleep onset latency (*β* = 1.01, 95% CI: 0.19–1.83, *p* = 0.02) and lower sleep efficiency (*β* = −0.60, 95% CI: −1.17 to −0.03, *p* = 0.04) were associated with lower weight loss. Higher WASO (*β* = 0.02, 95% CI: 0.00–0.05, *p* = 0.05) and increased sleep awakenings (*β* = 0.07, 95% CI: 0.02–0.13, *p* = 0.01) were associated with decreased weight loss at 18 months. Sleep duration and sleep regularity were not associated with weight loss.
Elder et al. (2012) [6]	Pre–post	Sleep duration Sleep quality	Insomnia Severity Index	Intervention: 6‐month behavioral weight loss intervention with weekly 90‐min group sessions. Target goals included reducing caloric intake by 500 kcal/day, following the Dietary Approaches to Stop Hypertension diet, and increasing physical activity to 180 min/week.	There was a significant quadratic trend between baseline sleep duration and weight loss success (OR = 0.80, 95% CI: 0.65–0.98, *p* = 0.030). Specifically, participants who slept > 6 and ≤ 8 h/night had higher odds of weight loss success (defined as weight loss of ≥ 4.5 kg throughout intervention), compared with participants who slept ≤ 6 or > 8 h/night. Sleep quality (measured using the total score of the Insomnia Severity Index) was not associated with weight loss or weight loss success.
Filiatrault et al. (2014) [39]	Pre–post	Sleep duration Sleep quality	PSQI	The control groups of four behavioral weight loss studies were pooled together in this analysis. In three of four studies, the control groups were instructed to reduce their daily caloric intake by 500–700 kcal/day. The control group in the remaining study underwent caloric restriction using Canada's Food Guide. Intervention lengths ranged from 12–16 weeks.	Participants who improved in sleep quality throughout the study experienced significantly higher reductions in waist circumference compared with participants who experienced deteriorations in sleep quality (improved sleep quality:‐5.8 cm, deteriorated sleep quality:‐3.7 cm, *p* < 0.05). Changes in sleep duration were not significantly associated with changes in weight loss indicators.
Garaulet et al. (2013) [38]	Pre–post	Sleep duration Chronotype	Self‐reported	Intervention: 20‐week behavioral weight loss intervention composed of weekly 60‐min group therapy sessions, with a reduction in caloric intake by 600–1100 kcal/day, physical activity of 15–30 min 2‐3x/week, and behavioral techniques such as stimulus control.	There was no significant association between sleep duration and chronotype with weight loss.
Hansen et al. (2019) [32]	Pre–post	Sleep duration Sleep quality Sleep variability	Sleep duration and sleep variability were measured using actigraphy. Sleep quality was measured using the PSQI.	Intervention: 8‐week behavioral weight loss intervention using a low‐energy diet of Modifast formula (5020 kJ/day for males and 4184 kJ/day for females).	A one‐unit increase in PSQI (lower sleep quality) was associated with −0.16 kg (95% CI: −0.02 to −0.30, *p* = 0.021) less weight loss. Sleep quality was not associated with changes in fat mass and fat‐free mass. No association was found between sleep duration and sleep variability with weight and adiposity loss.
Kline et al. (2021) [36]	Pre–post	Sleep duration Sleep efficiency Sleep quality Sleep timing Sleep regularity Daytime sleepiness	Sleep duration, sleep efficiency, sleep timing, and sleep regularity were measured using actigraphy. Sleep quality was measured using the PSQI. Daytime sleepiness was measured using the Epworth Sleepiness Scale.	Intervention: 12‐month behavioral weight loss intervention, including group sessions focused on diet and physical activity. Caloric restriction goals were given to achieve 7% body weight loss, and participants were instructed to partake in 150 min of moderate‐to‐vigorous physical activity per week.	When assessed as continuous variables, lower sleep quality (*p* = 0.042), worse sleep regularity (*p* = 0.005), and later sleep timing (*p* = 0.015) were all individually associated with decreased weight loss throughout the intervention. Low daytime sleepiness (Epworth Sleepiness Scale ≤ 10) was also associated with greater fat‐free mass loss (*p* = 0.024). Higher sleep regularity (*p* = 0.020), earlier sleep timing (*p* = 0.041), and increased sleep efficiency (*p* = 0.026) were also associated with greater percentage fat mass loss.
Mazri et al. (2023) [33]	Pre–post	Sleep duration Social jetlag	Munich Chronotype Questionnaire	Intervention: 12‐week behavioral weight loss intervention involving caloric restriction, weekly group physical activity sessions, and behavioral therapy.	No associations were found between changes in sleep duration or social jetlag and weight loss throughout the weight loss intervention.
Nielsen et al. (2021) [34]	Pre–post	Sleep duration Sleep variability	Actigraphy and sleep logs	Intervention: Surgical weight loss intervention including Roux‐en‐Y gastric bypass or sleeve gastrectomy. Participants were followed 3 months before surgery until 18 months after surgery.	Decreased sleep variability was associated with lower percentage fat‐free mass loss at 18 months postsurgery. There were no significant associations between sleep variability and sleep duration with other weight or adiposity loss indicators.
Sawamoto et al. (2014) [41]	Pre–post	Sleep duration Sleep latency Sleep efficiency Sleep disturbances Sleep quality	Sleep latency, sleep duration, sleep efficiency, and sleep disturbances were measured using actigraphy. Sleep quality was measured using the PSQI.	Intervention: 44‐week behavioral weight loss intervention composed of caloric restriction (reduction of 500 kcal/day), daily moderate physical activity, and cognitive behavioral therapy group sessions	A significant correlation was found between baseline sleep efficiency and reduction in both BMI (*r* = 0.271; *p* < 0.01) and body fat ratio (*r* = 0.26; *p* = 0.015). The number of wake episodes was also correlated with BMI (*r* = −0.35; *p* < 0.01) and body fat ratio (*r* = 0.24; *p* = 0.03). The number of wake episodes significantly predicted BMI reduction (*β* = − 0.341, *p* = 0.002). Sleep quality and sleep duration were not significantly correlated with BMI reduction.
Stromberg et al. (2020) [30]	Pre–post	Sleep duration Sleep quality	Medical Outcomes Study Sleep Scale (MOS‐Sleep)	Intervention: Surgical intervention involving Roux‐en‐Y gastric bypass or laparoscopic sleeve gastrectomy. Participants were followed from presurgery to 3 months postsurgery.	Both pre‐surgical sleep quality and sleep duration did not predict postsurgery percentage excess weight loss.
Swanson et al. (2025) [43]	Pre–post	Chronotype (sleep midpoint) Social jetlag Sleep duration Sleep loss (Weekly sleep debt)	Munich Chronotype Questionnaire	Intervention: 16‐week behavioral weight loss intervention, including weekly group sessions. This included personalized caloric deficit goals (1200–1800 kcal/day) and physical activity scaled up from 50 min of MVPA/week to 300 min/week.	No statistically significant associations were found between any of the sleep variables (chronotype, social jetlag, sleep duration, and sleep loss) and weight loss throughout the intervention.
Verhoef et al. (2013) [29]	Pre–post	Sleep duration	Self‐reported	Intervention: 8‐week behavioral weight loss intervention involving a very‐low‐energy diet of 2.1 megajoules/day using Modifast formula.	Change in sleep duration was negatively correlated with change in BMI following the weight loss intervention (*p* < 0.05) and at 3‐month follow‐up (*p* < 0.01). Change in sleep duration was also negatively correlated with change in fat mass at the 3‐month follow‐up (*p* < 0.05).
Wen et al. (2023) [35]	Pre–post	Sleep duration	Mi Band (actigraphy)	Intervention: 12‐week behavioral weight loss intervention including caloric restriction (daily energy intake of 20–25 kcal/kg) and physical activity (≥ 10,000 steps/day).	Participants were divided into sleep deprivation (< 6 h/night) and nonsleep deprivation (7–9 h/night) groups for analysis. Compared with the sleep deprivation group, the nonsleep deprivation group lost significantly more body weight (*t* = −2.86, *p* = 0.013) and more body fat content (t = −2.44, *p* = 0.029) following the weight loss intervention.

### 3.2 Risk of Bias

**FIGURE 2:**
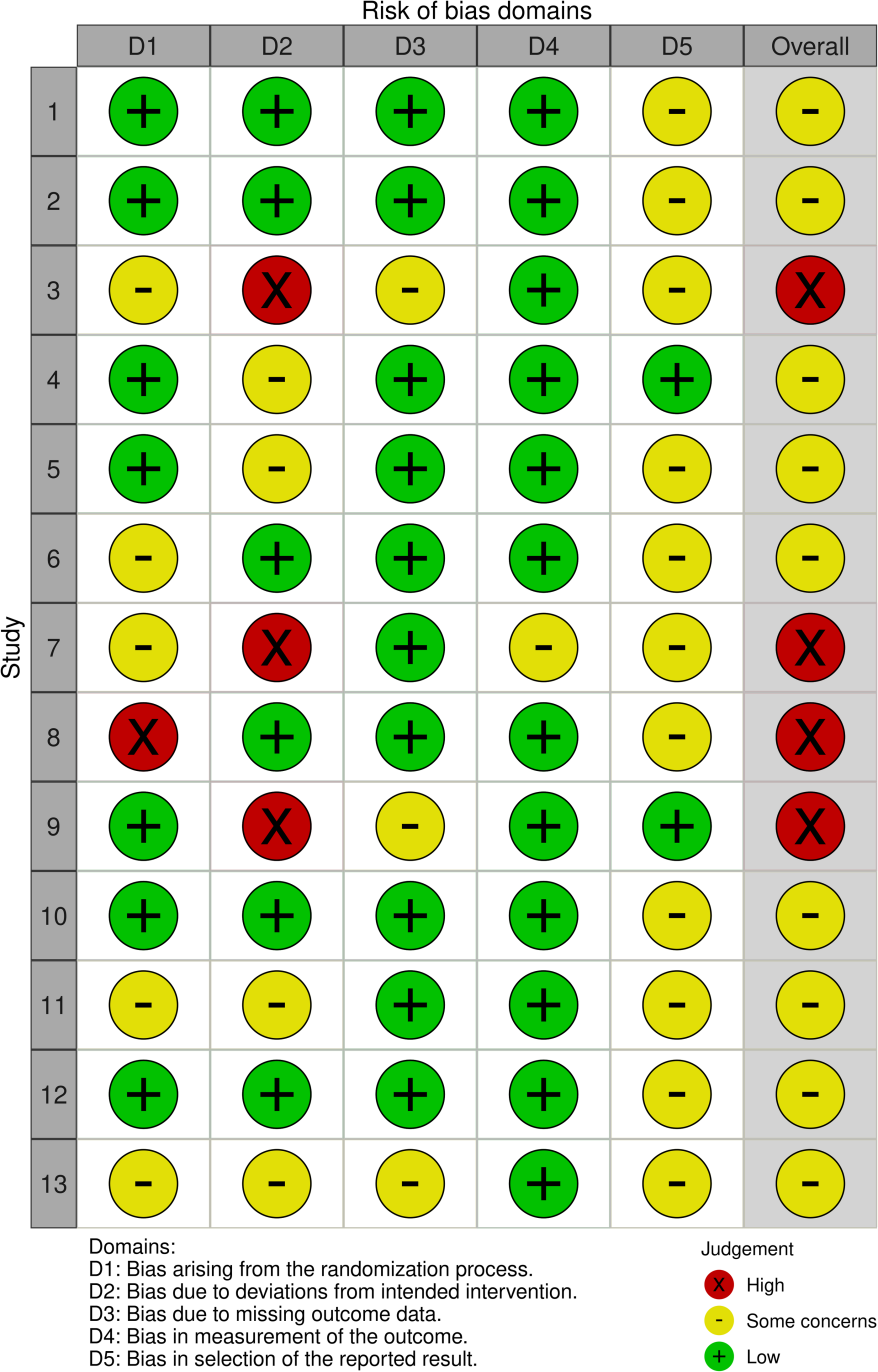
Traffic light plot of RoB‐2 assessments of randomized controlled trials (*n* = 11).

**FIGURE 3:**
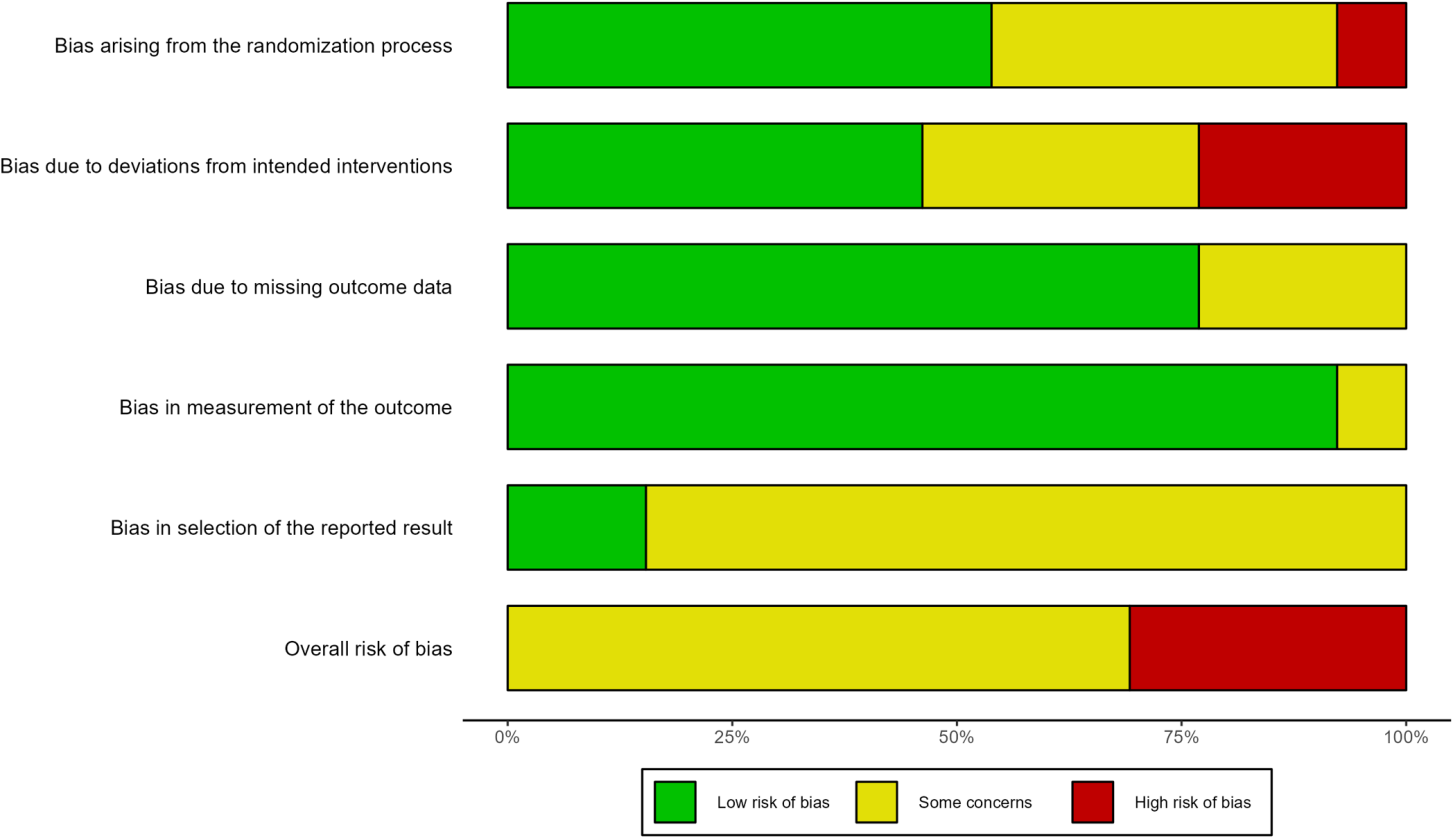
Summary bar plot of RoB‐2 assessments of randomized controlled trials (*n* = 11).

The Cochrane RoB‐2 tool was used to assess the risk of bias for RCTs. Overall, nine studies had some concerns about the risk of bias, and four studies were at high risk of bias (Figure 2). A summary of the risk of bias judgment in each domain is presented in Figure 3. Studies were classified as high risk of bias due to issues with Domain 1 (bias due to randomization) and Domain 2 (bias due to deviations from intended intervention). Domain 5 (bias due to selection of the reported results) was judged as having some concerns of risk of bias for 11 of 13 studies due to missing protocols or statistical analysis plans.

**FIGURE 4:**
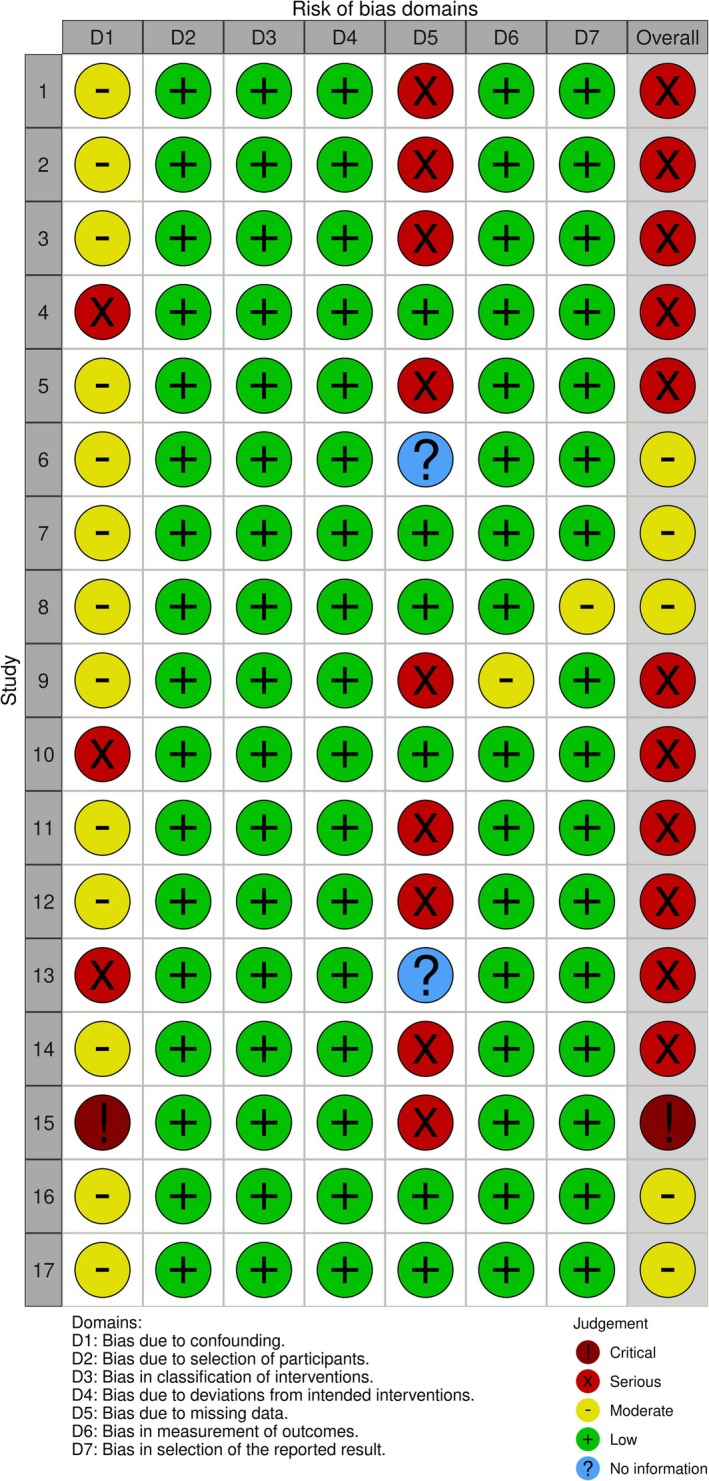
Traffic light plot of ROBINS‐I assessments of quasi‐experimental studies (*n* = 17).

**FIGURE 5:**
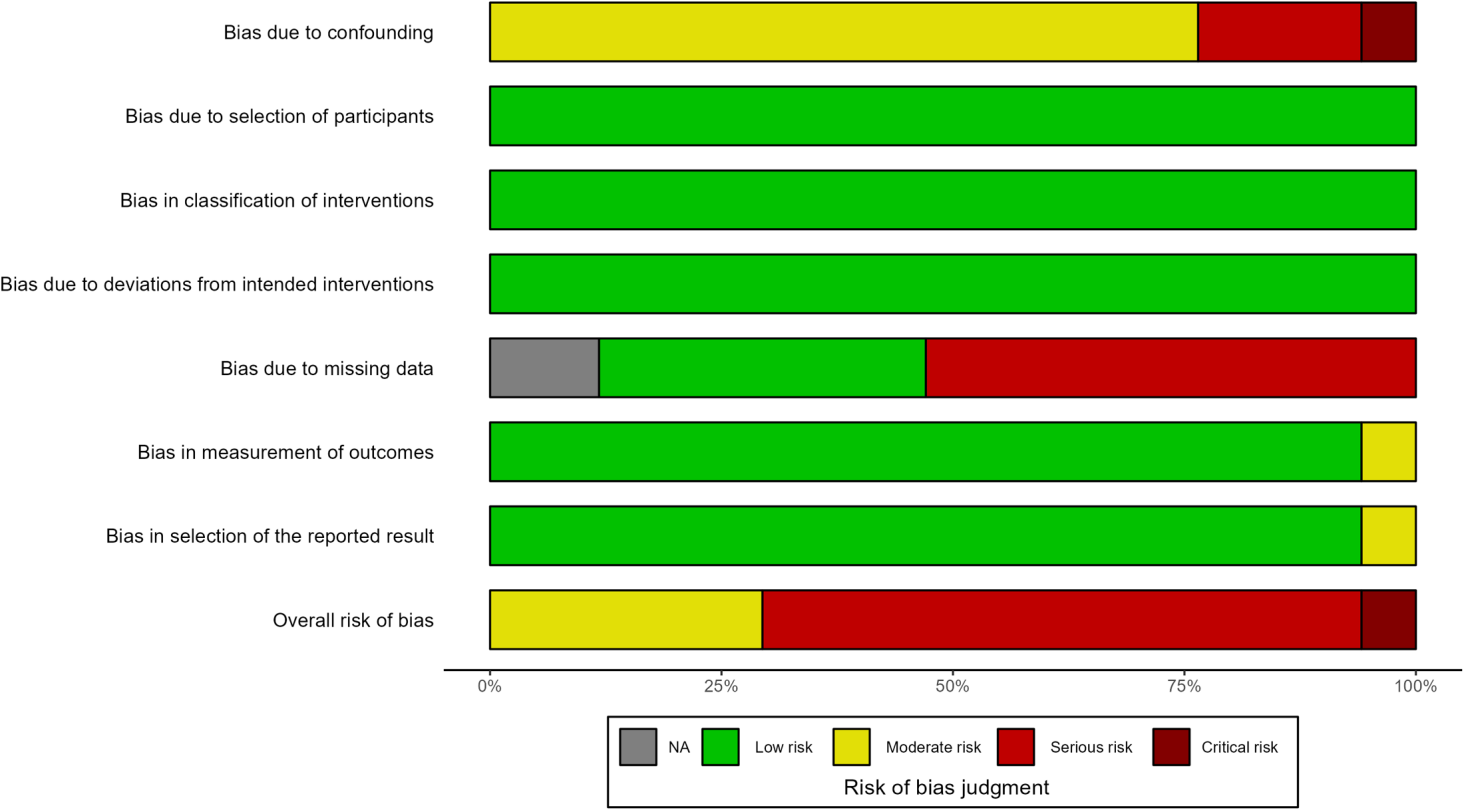
Summary bar plot of ROBINS‐I assessments of quasi‐experimental studies (*n* = 17).

The Cochrane ROBINS‐I tool was used to assess the risk of bias for nonrandomized studies of the effects of interventions. Five studies were considered to have moderate risk of bias, 11 studies were considered to have serious risk of bias, and one study was considered to have critical risk of bias (Figures 4 and 5). All studies were considered at least to have a moderate risk of bias due to the inherent risk of confounding. The majority of studies classified as serious risk of bias had over 15% missingness in the weight loss outcome, without appropriate analyses to account for missingness [29, 31, 37, 39, 40, 41, 44]. The study with critical risk of bias used *t*‐tests as its primary analysis, without accounting for any confounders in its findings [35]. Two studies were marked as “No information” for Domain 5 (bias due to missing data) as there was insufficient information reported to determine the degree of missingness in outcome data.

### 3.3 Sleep Duration

Two studies directly intervened on sleep duration, whereas 23 studies examined sleep duration as an exposure. Both RCTs using sleep restriction interventions demonstrated consistent reductions in fat mass loss in participants assigned to restricted sleep conditions, despite similar overall weight loss compared with control groups [18, 22]. For instance, Wang et al. [22] reported that the sleep restriction + caloric restriction group experienced a significantly lower total percentage of weight loss as fat (mean: 16.9%, SD: 104.5%) compared with the caloric restriction control group (mean: 80.7%, SD: 43.3%).

Among studies that did not intervene on sleep, eight RCTs and 15 quasi‐experimental studies examined the association between sleep duration and weight loss (Table 3) [6, 16, 17, 19, 20, 21, 26, 27, 28, 29, 30, 31, 32, 33, 34, 35, 36, 37, 38, 39, 41, 43, 44]. Sleep duration was measured by a mix of objective and subjective measures, including actigraphy, PSQI, and other questionnaires. Most studies employed behavioral weight loss interventions; however, four pre–post studies used bariatric surgery interventions [30, 31, 34, 44].

**TABLE 3:** High‐level summary of results by sleep health dimension.

Sleep health dimension	Number of studies	Key findings
Sleep duration	Behavioral weight loss interventions: Weight loss: RCTs (*n* = 10)	*n* = 1 study reported that longer sleep duration is associated with greater weight loss [28]. *n* = 7 studies reported null findings [16, 18, 19, 21, 22, 26, 27]. *n* = 2 studies reported that shorter sleep duration is associated with greater weight loss [17, 20].
	Pre–post studies (*n* = 11)	*n* = 1 study reported that longer sleep duration is associated with greater weight loss [35]. *n* = 8 studies reported null findings [32, 33, 36, 37, 38, 39, 41, 43].
	Adiposity loss: RCTs (*n* = 4) Pre–post studies (*n* = 6) Surgical weight loss interventions: Weight loss: Pre–post studies (*n* = 4) Adiposity loss: Pre–post studies (*n* = 1)	*n* = 1 study reported that shorter sleep duration is associated with greater weight loss [29]. *n* = 1 study reported a quadratic trend between sleep duration and weight loss [6]. *n* = 4 studies reported that longer sleep duration is associated with greater adiposity loss [16, 18, 19, 22]. *n* = 2 studies reported that longer sleep duration is associated with greater adiposity loss [35, 40]. *n* = 3 studies reported null findings [32, 36, 39]. *n* = 1 study reported that shorter sleep duration is associated with greater adiposity loss [29]. *n* = 4 studies reported null findings [30, 31, 34, 44]. *n* = 1 study reported null findings [34].
Sleep satisfaction	Behavioral weight loss interventions: Weight loss: RCTs (*n* = 4)	*n* = 2 studies reported that better sleep quality is associated with greater weight loss [16, 20]. *n* = 1 study reported null findings [23]. *n* = 1 study reported that lower sleep quality is associated with greater weight loss [17].
	Pre–post studies (*n* = 5)	*n* = 3 studies reported that better sleep quality is associated with greater weight loss [32, 36, 39]. *n* = 2 studies reported null findings [6, 41].
	Adiposity loss: RCTs (*n* = 1) Pre–post studies (*n* = 4) Surgical weight loss interventions: Weight loss: Pre–post studies (*n* = 2)	*n* = 1 study reported null findings [16]. *n* = 1 study reported that better sleep quality is associated with greater adiposity loss [40]. *n* = 3 studies reported null findings [32, 36, 41]. *n* = 1 study reported that better sleep quality is associated with greater weight loss [42]. *n* = 1 study reported null findings [30].
Sleep efficiency	Behavioral weight loss interventions: Weight loss: RCTs (*n* = 9)	*n* = 5 studies reported that higher sleep efficiency is associated with greater weight loss [20, 24, 25, 26, 27]. *n* = 4 studies reported null findings [16, 17, 19, 28].
	Pre–post studies (*n* = 3) Adiposity loss: RCTs (*n* = 2) Pre–post studies (*n* = 1)	*n* = 2 studies reported that higher sleep efficiency is associated with greater weight loss [37, 41]. *n* = 1 study reported null findings [36]. *n* = 1 study reported that higher sleep efficiency is associated with greater adiposity loss [24]. *n* = 1 study reported null findings [16]. *n* = 1 reported that higher sleep efficiency is associated with greater adiposity loss [36].
Sleep regularity	Behavioral weight loss interventions: Weight loss: RCTs (*n* = 1) Pre–post studies (*n* = 5) Adiposity loss: Pre–post studies (*n* = 2) Surgical weight loss interventions: Weight loss: Pre–post studies (*n* = 2) Adiposity loss: Pre–post studies (*n* = 1)	*n* = 1 study reported that higher sleep regularity is associated with greater weight loss [19]. *n* = 1 study reported that higher sleep regularity is associated with greater weight loss [36]. *n* = 4 studies reported null findings [32, 33, 37, 43]. *n* = 1 study reported that higher sleep regularity is associated with greater adiposity loss [36]. *n* = 1 study reported null findings [32]. *n* = 1 study reported that higher sleep regularity is associated with greater weight loss [42]. *n* = 1 study reported null findings [34]. *n* = 1 study reported null findings [34].
Sleep timing	Behavioral weight loss interventions: Weight loss: RCTs (*n* = 2)	*n* = 1 study reported that both an earlier rise time and later bedtime are associated with greater weight loss [27]. *n* = 1 study reported null findings [19].
	Pre–post studies (*n* = 4)	*n* = 1 reported that earlier sleep timing is associated with greater weight loss [36]. *n* = 3 studies reported null findings [37, 38, 43].
	Adiposity loss: Pre–post studies (*n* = 1) Surgical weight loss interventions: Weight loss: Pre–post studies (*n* = 1)	*n* = 1 reported that earlier sleep timing is associated with greater adiposity loss [36]. *n* = 1 study reported that earlier sleep timing is associated with greater weight loss [31].
Alertness	Behavioral weight loss interventions: Weight loss: RCTs (*n* = 3) Pre–post studies (*n* = 1) Adiposity loss: Pre–post studies (*n* = 1) Surgical weight loss interventions: Weight loss: Pre–post studies (*n* = 1)	*n* = 3 studies reported null findings [17, 20, 27]. *n* = 1 study reported null findings [36]. *n* = 1 study reported null findings [36]. *n* = 1 study reported that higher alertness is associated with greater weight loss [42].

Among RCTs with behavioral weight loss interventions consisting of caloric restriction and/or physical activity, one study reported an association between longer sleep duration and greater weight loss [28], two studies reported associations between shorter sleep duration and greater weight loss [17, 20], and five studies reported null findings [16, 19, 21, 26, 27].

Among quasi‐experimental studies with behavioral weight loss interventions, one study found that longer sleep duration was associated with greater weight loss [35], whereas eight studies reported null findings [32, 33, 36, 37, 38, 39, 41, 43]. Conversely, one study reported that decreased sleep duration over time was associated with greater weight loss following the weight loss intervention (*p* < 0.05) and at 3‐month follow‐up (*p* < 0.01) [29]. In addition, Elder et al. identified a significant quadratic trend between baseline sleep duration and weight loss success (OR = 0.80, 95% CI: 0.65–0.98, *p* = 0.030) [6]. Specifically, participants who slept between 6 and 8 h/night had higher odds of weight loss success (weight loss of ≥ 4.5 kg) after the weight loss intervention, compared with participants who slept ≤ 6 or > 8 h/night. All four pre–post studies with bariatric surgery interventions found no association between sleep duration and weight loss [30, 31, 34, 44].

Among studies that examined sleep duration as an exposure, two RCTs and seven pre–post studies reported on the relationship between sleep duration and adiposity loss. The 2023 RCT from Bogh et al. reported that longer sleep duration was associated with greater reductions in body fat (*β* = −0.80, *p* = 0.026) [16]. On a similar note, Papandreou et al. [19] found that shorter sleep duration was associated with lower waist circumference reduction (*β* = −0.03, *p* = 0.024). Within pre–post studies, two studies reported associations between higher sleep duration and greater body fat loss [35, 40], four studies reported null findings (including one study with a bariatric surgery intervention) [32, 34, 36, 39], and one study reported that increased sleep duration over time was associated with greater fat mass loss (*p* < 0.05) [29].

### 3.4 Sleep Satisfaction

Four RCTs and seven pre–post studies examined the association between sleep satisfaction and weight loss throughout a weight loss intervention [6, 16, 17, 20, 23, 30, 32, 36, 39, 41, 42]. In most studies, sleep satisfaction was measured by self‐report questionnaires such as the PSQI. Almost all studies employed a behavioral weight loss intervention, consisting of a combination of caloric restriction, physical activity, and/or counseling, whereas two pre–post studies used a bariatric surgery intervention [30, 42].

Among RCTs, two studies reported a statistically significant association between higher sleep satisfaction and greater weight loss [16, 20], and one study reported null findings [23]. In addition, Littman et al. [17] reported that, compared with the control group, participants reporting poorer sleep satisfaction experienced a greater difference in weight loss following the 1‐year physical activity intervention (difference: −3.6 kg, 95% CI: −7.2 to 0.1 kg). However, this association was not statistically significant, and the authors suggested it may have been influenced by weight gain in the control group.

Among pre–post studies with behavioral weight loss interventions, three studies reported a statistically significant association between higher sleep satisfaction and greater weight loss [32, 36, 39]. Two studies found no association between sleep satisfaction and weight loss following the weight loss intervention [6, 41]. The two studies using bariatric surgery interventions reported conflicting findings; one reported null findings [30], whereas Carvalho et al. [42] documented that higher sleep satisfaction (lower PSQI score) was associated with greater total weight loss at 3 months, 6 months, and 1 year postsurgery (3 months: *β* = −0.22; *p* = 0.001, 6 months: *β* = −0.33; *p* < 0.001, 1 year: *β* = −0.33; *p* = 0.002).

One RCT and four pre–post studies reported on how sleep satisfaction was related to adiposity loss. All studies reported null findings [16, 32, 36, 41], except for the pre–post study by Chaput and Tremblay [40], which reported that lower sleep satisfaction (higher PSQI score) was linked to lower fat mass loss (*β* = −0.19, *p* < 0.05) and lower percentage body fat loss (*β* = −0.21, *p* < 0.01).

### 3.5 Sleep Efficiency

Nine RCTs and three pre–post studies examined the association between sleep efficiency and weight loss [16, 17, 19, 20, 24, 25, 26, 27, 28, 29, 36, 37, 41]. Sleep efficiency was mainly measured using the PSQI or actigraphy, and indicators of sleep efficiency included percentage sleep efficiency, wake after sleep onset, rise time latency, sleep onset latency, and sleep disturbances. All studies used a variation of a behavioral weight loss intervention.

Among RCTs, five studies observed a statistically significant association between better sleep efficiency and greater weight loss [20, 24, 25, 26, 27]. Four RCTs did not show any significant association between sleep efficiency and weight loss by the end of the intervention [16, 17, 19, 28].

Of the three pre–post studies, two studies reported an association between higher sleep efficiency and greater weight loss following the behavioral weight loss intervention [37, 41], whereas Kline et al. [36] reported null findings.

Two RCTs and one pre–post study also reported on the relationship between sleep efficiency and adiposity loss. One RCT reported null findings [16], whereas another RCT observed a statistically significant association between lower sleep efficiency and lower reductions in total fat loss (*p* = 0.02), total fat mass percentage (*p* = 0.04), and trunk fat percentage (*p* = 0.05) [24]. The pre–post study reported a statistically significant association between higher sleep efficiency and greater percentage fat mass loss (*p* = 0.026) [36].

### 3.6 Sleep Regularity

One RCT and seven pre–post studies investigated the association between sleep regularity and weight loss. Sleep regularity was measured mostly using actigraphy or self‐reported questionnaires. Studies measured different indicators of sleep regularity, including sleep regularity, sleep variability, and social jetlag. Higher variability and greater social jetlag represent lower sleep regularity and more circadian misalignment. Most studies deployed a behavioral weight loss intervention, whereas two studies used surgical interventions [34, 42].

An RCT conducted by Papandreou et al. [19] reported that participants belonging to the low sleep regularity (high sleep variability) group experienced less weight loss (*p* = 0.020) and lower reductions in BMI at 12 months (*p* = 0.016) compared with the high sleep regularity (low sleep variability) group.

Among pre–post studies with behavioral weight loss interventions, Kline et al. [36] reported a statistically significant association between higher sleep regularity (*p* = 0.005) and greater weight loss. Meanwhile, four studies reported null findings [32, 33, 37, 43]. Among studies with surgical interventions, Carvalho et al. [42] noted that lower sleep regularity (higher social jetlag) was associated with decreased total weight loss (kg) at 6 months (*β* = −0.14, *p* = 0.03) and 1 year postsurgery (*β* = −0.24, *p* = 0.03). In addition, Nielsen et al. [34] did not find any associations between sleep regularity and weight loss.

In total, three pre–post studies examined the associations between sleep regularity and adiposity loss. One study reported a positive association between sleep regularity and percentage fat mass loss (*p* = 0.020) [36], and another reported null findings [32]. Nielsen et al. [34] reported that higher sleep regularity (decreased sleep variability) over time was associated with lower percentage fat‐free mass loss at 18 months following bariatric surgery (*p* = 0.03).

### 3.7 Sleep Timing

Two RCTs and five pre–post studies examined the association between sleep timing and weight loss [19, 27, 31, 36, 37, 38, 43]. Sleep timing was measured either using actigraphy or self‐reported sleep and wake times, and assessed as bedtime and wake time or phase delay. All studies used behavioral weight loss interventions, except for one pre–post study, which used a bariatric surgery intervention [31].

Across the included RCTs, Altree et al. [27] reported that later bedtime at baseline was correlated with greater weight loss (rho = −0.45, *p* < 0.001) following a 2‐month very low energy dietary intervention. In contrast, participants who adopted earlier rise times throughout the intervention (mean change in rise time: −12.6 ± −45.3 min) experienced significantly greater weight loss (rho = 0.36, *p* = 0.03). The other RCT reported no statistically significant association between sleep timing and weight loss [19].

Among pre–post studies with behavioral weight loss interventions, three studies reported null findings between sleep timing and weight loss [37, 38, 43]. Another pre–post study found that earlier sleep timing was associated with increased weight loss (*p* = 0.015) and percentage fat mass loss (*p* = 0.041) throughout the caloric restriction and physical activity intervention [36]. Similarly, the bariatric surgery study from Barnadas‐Sole et al. [31] reported that participants with later bedtimes (≥ 24:00 h) lost 5.64% less excess weight (95% CI: −10.11%, −1.17%) compared with early sleepers (< 24:00 h).

### 3.8 Alertness

Three RCTs and two pre–post studies explored the relationship between alertness and weight loss [17, 20, 27, 36, 42]. In all studies, alertness was assessed as daytime sleepiness using questionnaires such as the Epworth Sleepiness Scale. Alertness was operationalized as daytime sleepiness (inverse of alertness). All studies employed a behavioral weight loss intervention except for one surgical pre–post study [42].

All three RCTs concluded that there was no statistically significant association between alertness and weight loss following the weight loss intervention [17, 20, 27].

Among pre–post studies, one study reported that low daytime sleepiness (high alertness) was not associated with any weight loss outcomes; however, it was associated with higher fat‐free mass loss (*p* = 0.024) [36]. In addition, Carvalho et al. [42] noted a statistically significant association between higher daytime sleepiness (lower alertness) and greater weight loss (*β* = −0.28, *p* = 0.009), percentage weight loss (*β* = −0.37, *p* = 0.002), and BMI change (*β* = −0.35, *p* = 0.002) at 1‐year following bariatric surgery.

## 4 Discussion

In this systematic review, we examined the current body of RCTs and quasi‐experimental studies regarding sleep health dimensions and their association with weight and adiposity loss in adults with excess weight undergoing a weight loss intervention. A total of 30 studies were included in this review, with 10,944 participants across 13 countries. Overall, the evidence base suggests heterogeneous and dimension‐specific associations between sleep health and weight loss outcomes. Sleep manipulation trials provide some evidence that insufficient sleep duration may impair fat loss during weight loss interventions, even when total weight loss between intervention groups is similar. In contrast, studies that did not intervene on sleep show inconsistent associations between sleep duration and weight loss, with no clear dose–response relationship. For other sleep health dimensions, including sleep satisfaction and sleep efficiency, there is limited but suggestive evidence of beneficial associations with weight loss in behavioral interventions, although findings are not uniform across studies. Preliminary findings suggest potential relevance of sleep regularity and sleep timing for weight loss success, whereas alertness does not appear to affect weight loss; however, all three sleep health domains have insufficient evidence to draw firm conclusions.

Among behavioral weight loss intervention studies, the evidence supporting the relationship between sleep duration and weight loss was largely mixed, with 15/21 (71%) of studies reporting no significant association [16, 19, 21, 26, 27, 32, 33, 36, 37, 38, 39, 41, 43]. Both sleep manipulation trials in our review reported null findings [18, 22], whereas the remaining studies that examined sleep duration as an exposure showed highly inconsistent results. Our results differ slightly from existing literature; a scoping review by Knowlden et al. [48] reported that higher baseline sleep duration was associated with increased weight loss from behavioral weight loss interventions; however, in studies where only physical activity interventions were given, higher baseline sleep duration was associated with decreased weight loss [17, 49]. Although this raises questions regarding how physical activity impacts the relationship between sleep duration and weight loss, we did not observe any differences in our findings by intervention type.

Approximately half of the studies (56%) using a behavioral weight loss intervention reported a positive association between sleep satisfaction and weight loss [16, 20, 32, 36, 39], providing limited evidence that better sleep satisfaction was associated with greater weight loss among adults with excess weight. This association remained consistent regardless of the sleep measurement method or the type of weight loss intervention. Our findings align with previous scoping and narrative reviews, which have reported modest evidence supporting a positive association between sleep satisfaction and weight loss [48, 50].

Previous studies have provided insight into the mechanisms by which sleep duration and sleep satisfaction could affect obesity and weight loss/gain. Inadequate sleep duration and poor sleep satisfaction are hypothesized to affect obesity through a variety of mechanisms, such as hormonal dysregulation, energy expenditure, and behavioral changes [51, 52]. Sleep restriction studies have shown that partial sleep deprivation is often associated with the consumption of more calorie‐dense and high‐fat diets compared with adequate sleep duration. Poor sleep satisfaction may also lead to increased cortisol levels and abdominal weight gain [51, 53].

Among behavioral weight loss intervention studies that examined sleep efficiency, 7/12 (58%) reported a positive association [20, 24, 25, 26, 27, 37, 41], indicating that there is some evidence to support that higher sleep efficiency is related to greater weight loss. Between studies, sleep efficiency was measured using a variety of indicators such as percentage sleep efficiency, sleep disturbances, wake after sleep onset, and sleep onset latency. Although all the aforementioned indicators were summarized as sleep efficiency for the purposes of this review, future research is needed to determine whether certain sleep efficiency indicators have more impact on weight loss than others. In addition, there was limited evidence supporting a relationship between sleep regularity and sleep timing with weight loss, and no relationship between alertness and weight loss. Across studies, lower sleep regularity was generally associated with reduced weight loss or attenuated fat loss. Findings also suggested that later or more irregular sleep timing may be associated with less favorable weight loss outcomes in some studies, although results were inconsistent. Notably, studies reporting associations between earlier wake times and greater weight loss [31, 36, 42] are directionally consistent with the hypothesis that earlier and more stable circadian timing supports better weight loss outcomes [10, 51]. However, the limited number of studies across these sleep health dimensions is insufficient to draw firm conclusions.

The body of research examining the relationship between sleep health dimensions and adiposity loss was largely inconclusive. In behavioral weight loss intervention studies, there was limited evidence suggesting that longer sleep duration may be associated with greater adiposity loss, although overall findings were mixed. Other sleep health dimensions were examined in only a few studies, most of which reported null results.

Among studies using bariatric surgery interventions, such as gastric bypass and sleeve gastrectomy, there was insufficient evidence to determine the association between sleep health dimensions and weight or adiposity loss. Only five surgical intervention studies were included in this review [30, 31, 34, 42, 44], and each sleep health dimension was assessed in just one or two studies. Within this limited evidence, better sleep satisfaction, higher sleep regularity, earlier sleep timing, and greater alertness were associated with increased weight loss; however, these findings should be interpreted with caution.

Inconsistent findings between and within sleep health dimensions may be attributed to inter‐individual variability. The relationship between sleep and weight loss is complex, and the direction of findings can depend on several biological and behavioral factors. For instance, a review of genome‐wide association studies has shown that certain loci may have an effect on both sleep and weight loss success, meaning that different individuals may be more genetically disposed or resistant to sleep‐related weight loss [54]. Existing comorbidities may also influence the effectiveness of sleep on weight loss; for example, obstructive sleep apnea is highly prevalent among adults with overweight or obesity [55], and it is characterized by deteriorations in sleep satisfaction and sleep efficiency. Lastly, behavioral factors such as caffeine intake, which negatively affects sleep health and increases energy expenditure [56, 57], can play a role in individual weight loss success. As many of our included studies did not account for all these factors in their analyses, these factors may explain some of the variability within our findings.

One strength of our study is the use of a comprehensive search strategy conducted with the aid of a research librarian. In addition, screening, data extraction, and risk of bias assessment were piloted and carried out in duplicate to ensure accuracy and reliability. However, our study possesses many limitations. First, a meta‐analysis was unable to be conducted due to clinical and methodological heterogeneity, specifically the type of weight loss interventions, length of the intervention, methods of sleep measurement, and statistical tests conducted. As a result, pre‐planned subgroup and sensitivity analyses were not completed. In addition, only studies in English or French were included in our review. However, a recent meta‐epidemiological study demonstrated that excluding non‐English publications from systematic reviews does not impact findings [58]. Our narrative synthesis, which groups studies by sleep health dimension, study design, and type of weight loss intervention, looks at the overarching direction of effect in each subgroup. As a result, this method may overlook the context and complexities of each study. As no quantitative analysis was conducted, we also could not appropriately weigh the results of different studies based on sample size and/or risk of bias.

Findings from our study have important implications for public health and policy. Our findings suggest that including sleep health in obesity programming in addition to physical activity and healthy dietary practices could be beneficial to support weight loss. In clinical settings that offer obesity management services, adults with excess weight should be provided with education and support on the importance of sleep health. However, further research is needed to address the uncertainty of our findings, particularly due to the lack of high‐quality experimental studies that examine the relationship between sleep health and weight/adiposity loss in adults with excess weight. In future studies, protocols and/or statistical analysis plans should be registered prior to study conduct, randomization methods should be explicitly stated when applicable, objective sleep measures should be used when feasible, intention‐to‐treat analyses should be prioritized over/in addition to per‐protocol analyses, and missing data should be addressed using appropriate statistical procedures. Future research should also focus on less frequently studied sleep health dimensions like sleep regularity, sleep timing, and daytime alertness. Additional studies using surgical weight loss interventions are also needed, and the relationship between sleep health and adiposity loss specifically should be further explored.

This systematic review also expands upon prior narrative work [48, 50, 59] by providing a structured synthesis using PRISMA methods, formal risk of bias assessment (RoB2 and ROBINS‐I), and explicit stratification by both sleep health domain and intervention type. Unlike previous narrative reviews, our approach allows clearer identification of methodological limitations and evidence gaps across sleep health domains.

## 5 Conclusion

In summary, our systematic review demonstrates that there is some evidence to support the role of sleep satisfaction and sleep efficiency in the effectiveness of behavioral weight loss interventions in adults with excess weight. Evidence from sleep manipulation trials suggests that insufficient sleep duration may impair fat loss. Among behavioral weight loss intervention studies, findings were mixed for sleep duration and weight loss, with limited evidence supporting the relationship between improved sleep regularity and sleep timing and greater weight loss. No associations were found between alertness and weight loss. The evidence on bariatric surgery interventions was inconclusive due to the sparse literature within these research areas. High‐quality experimental studies are needed for all sleep health dimensions to further confirm our findings.

## Author Contributions

J.‐P.C. and L.D. conceptualized the systematic review. L.D., J.B., N.D., and G.S. screened the articles. J.B. and G.S. extracted the data. L.D. and N.D. conducted the risk of bias assessment. L.D., J.B., N.D., and G.S. prepared the first manuscript draft, with supervision and feedback from J.‐P.C. All authors critically revised the manuscript for important intellectual content. All authors take final responsibility for the decision to submit the article for publication.

## Funding

The authors have nothing to report.

## Conflicts of Interest

The authors declare no conflicts of interest.

## Supporting information




**Data S1:** Supporting Information.

## Data Availability

Data sharing not applicable to this article as no datasets were generated or analysed during the current study.

## References

[1] G. Jean‐Louis , N. J. Williams , D. Sarpong , et al., “Associations Between Inadequate Sleep and Obesity in the US Adult Population: Analysis of the National Health Interview Survey (1977–2009),” BMC Public Health 14, no. 1 (2014): 290, 10.1186/1471-2458-14-290. 24678583 PMC3999886

[2] J. C. Seidell and J. Halberstadt , “The Global Burden of Obesity and the Challenges of Prevention,” Annals of Nutrition & Metabolism 66, no. Suppl. 2 (2015): 7–12, 10.1159/000375143. 26045323

[3] “ICD‐11 for Mortality and Morbidity Statistics,” Accessed February 24, 2024, https://icd.who.int/browse/2024‐01/mms/en.

[4] C. Boutari and C. S. Mantzoros , “A 2022 Update on the Epidemiology of Obesity and a Call to Action: As Its Twin COVID‐19 Pandemic Appears to Be Receding, the Obesity and Dysmetabolism Pandemic Continues to Rage on,” Metabolism 133 (2022): 155217, 10.1016/j.metabol.2022.155217. 35584732 PMC9107388

[5] L. Viester , E. A. Verhagen , K. M. O. Hengel , L. L. Koppes , A. J. Van Der Beek , and P. M. Bongers , “The Relation Between Body Mass Index and Musculoskeletal Symptoms in the Working Population,” BMC Musculoskeletal Disorders 14, no. 1 (2013): 238, 10.1186/1471-2474-14-238. 23937768 PMC3751130

[6] C. R. Elder , C. M. Gullion , K. L. Funk , L. L. DeBar , N. M. Lindberg , and V. J. Stevens , “Impact of Sleep, Screen Time, Depression and Stress on Weight Change in the Intensive Weight Loss Phase of the LIFE Study,” International Journal of Obesity 36, no. 1 (2012): 86–93, 10.1038/ijo.2011.60. 21448129 PMC3136584

[7] Y. Wu , L. Zhai , and D. Zhang , “Sleep Duration and Obesity Among Adults: A Meta‐Analysis of Prospective Studies,” Sleep Medicine 15, no. 12 (2014): 1456–1462, 10.1016/j.sleep.2014.07.018. 25450058

[8] S. R. Patel and F. B. Hu , “Short Sleep Duration and Weight Gain: A Systematic Review,” Obesity 16, no. 3 (2008): 643–653, 10.1038/oby.2007.118. 18239586 PMC2723045

[9] D. J. Buysse , “Sleep Health: Can We Define It? Does It Matter?,” Sleep 37, no. 1 (2014): 9–17, 10.5665/sleep.3298. 24470692 PMC3902880

[10] J. P. Chaput , A. W. McHill , R. C. Cox , et al., “The Role of Insufficient Sleep and Circadian Misalignment in Obesity,” Nature Reviews. Endocrinology 19, no. 2 (2023): 82–97, 10.1038/s41574-022-00747-7. PMC9590398 36280789

[11] G. Beccuti and S. Pannain , “Sleep and Obesity: Current Opinion in Clinical Nutrition and Metabolic Care,” 14, no. 4 (2011): 402–412, 10.1097/MCO.0b013e3283479109. PMC3632337 21659802

[12] M. J. Page , J. E. McKenzie , P. M. Bossuyt , et al., “The PRISMA 2020 Statement: An Updated Guideline for Reporting Systematic Reviews,” Systematic Reviews 10, no. 1 (2020): 89, 10.1186/s13643-021-01626-4. PMC8008539 33781348

[13] C. Schardt , M. B. Adams , T. Owens , S. Keitz , and P. Fontelo , “Utilization of the PICO Framework to Improve Searching PubMed for Clinical Questions,” BMC Medical Informatics and Decision Making 7, no. 1 (2007): 16, 10.1186/1472-6947-7-16. 17573961 PMC1904193

[14] J. McGowan , M. Sampson , D. M. Salzwedel , E. Cogo , V. Foerster , and C. Lefebvre , “PRESS Peer Review of Electronic Search Strategies: 2015 Guideline Statement,” Journal of Clinical Epidemiology 75 (2016): 40–46, 10.1016/j.jclinepi.2016.01.021. 27005575

[15] J. R. Polanin , T. D. Pigott , D. L. Espelage , and J. K. Grotpeter , “Best Practice Guidelines for Abstract Screening Large‐Evidence Systematic Reviews and Meta‐Analyses,” Research Synthesis Methods 10, no. 3 (2019): 330–342, 10.1002/jrsm.1354.

[16] A. F. Bogh , S. B. K. Jensen , C. R. Juhl , et al., “Insufficient Sleep Predicts Poor Weight Loss Maintenance After 1 Year,” Sleep 46, no. 5 (2023): zsac295, 10.1093/sleep/zsac295. 36472579 PMC10171640

[17] A. J. Littman , M. V. Vitiello , K. Foster‐Schubert , et al., “Sleep, Ghrelin, Leptin and Changes in Body Weight During a 1‐Year Moderate‐Intensity Physical Activity Intervention,” International Journal of Obesity 31, no. 3 (2007): 466–475, 10.1038/sj.ijo.0803438. 16909130

[18] A. V. Nedeltcheva , J. M. Kilkus , J. Imperial , D. A. Schoeller , and P. D. Penev , “Insufficient Sleep Undermines Dietary Efforts to Reduce Adiposity,” Annals of Internal Medicine 153, no. 7 (2010): 435–441, 10.7326/0003-4819-153-7-201010050-00006. 20921542 PMC2951287

[19] C. Papandreou , M. Bulló , A. Díaz‐López , et al., “High Sleep Variability Predicts a Blunted Weight Loss Response and Short Sleep Duration a Reduced Decrease in Waist Circumference in the PREDIMED‐Plus Trial,” International Journal of Obesity 44, no. 2 (2020): 330–339, 10.1038/s41366-019-0401-5. 31217539

[20] C. A. Thomson , K. L. Morrow , S. W. Flatt , et al., “Relationship Between Sleep Quality and Quantity and Weight Loss in Women Participating in a Weight‐Loss Intervention Trial,” Obesity 20, no. 7 (2012): 1419–1425, 10.1038/oby.2012.62. 22402738 PMC4861065

[21] H. Tuomilehto , M. Peltonen , M. Partinen , et al., “Sleep Duration, Lifestyle Intervention, and Incidence of Type 2 Diabetes in Impaired Glucose Tolerance: The Finnish Diabetes Prevention Study,” Diabetes Care 32, no. 11 (2009): 1965–1971, 10.2337/dc08-1980. 19651919 PMC2768215

[22] X. Wang , J. R. Sparks , K. P. Bowyer , and S. D. Youngstedt , “Influence of Sleep Restriction on Weight Loss Outcomes Associated With Caloric Restriction,” Sleep 41, no. 5 (2018): zsy027, 10.1093/sleep/zsy027. PMC8591680 29438540

[23] T. Fritz , K. Caidahl , M. Osler , C. G. Östenson , J. R. Zierath , and P. Wändell , “Effects of Nordic Walking on Health‐Related Quality of Life in Overweight Individuals With Type 2 Diabetes Mellitus, Impaired or Normal Glucose Tolerance,” Diabetic Medicine 28, no. 11 (2011): 1362–1372, 10.1111/j.1464-5491.2011.03348.x. 21658122 PMC3229676

[24] A. Li , X. Li , T. Zhou , et al., “Sleep Disturbance and Changes in Energy Intake and Body Composition During Weight Loss in the POUNDS Lost Trial,” Diabetes 71, no. 5 (2022): 934–944, 10.2337/db21-0699. 35202470 PMC9044134

[25] L. G. Rosas , K. M. J. Azar , N. Lv , et al., “Effect of an Intervention for Obesity and Depression on Patient‐Centered Outcomes: An RCT,” American Journal of Preventive Medicine 58, no. 4 (2020): 496–505, 10.1016/j.amepre.2019.11.005. 32067873 PMC7089815

[26] E. M. O'Brien , J. Fava , L. L. Subak , et al., “Sleep Duration and Weight Loss Among Overweight/Obese Women Enrolled in a Behavioral Weight Loss Program,” Nutrition & Diabetes 2, no. SEPTEMBER (2012): e43, 10.1038/nutd.2012.17. 23446658 PMC3461353

[27] T. J. Altree , D. J. Bartlett , N. S. Marshall , et al., “Predictors of Weight Loss in Obese Patients With Obstructive Sleep Apnea,” Sleep and Breathing 26, no. 2 (2022): 753–762, 10.1007/s11325-021-02455-4. 34357505

[28] C. I. R. Braem , W. J. Pasman , T. J. van den Broek , et al., “The Association of Physical Activity, Heart Rate and Sleep From an Activity Tracker With Weight Loss During a 6‐Month Personalized Combined Lifestyle Intervention: A Retrospective Analysis,” BMC Digital Health 3, no. 1 (2025): 8, 10.1186/s44247-024-00145-1.

[29] S. P. Verhoef , S. G. Camps , H. K. Gonnissen , K. R. Westerterp , and M. S. Westerterp‐Plantenga , “Concomitant Changes in Sleep Duration and Body Weight and Body Composition During Weight Loss and 3‐Mo Weight Maintenance123,” American Journal of Clinical Nutrition 98, no. 1 (2013): 25–31, 10.3945/ajcn.112.054650. 23697706

[30] S. E. Stromberg , R. Gonzalez‐Louis , M. Engel , A. Mathews , and D. M. Janicke , “Pre‐Surgical Stress and Social Support Predict Post‐Surgical Percent Excess Weight Loss in a Population of Bariatric Surgery Patients,” Psychology, Health & Medicine 25, no. 10 (2020): 1258–1265, 10.1080/13548506.2020.1734216. 32101050

[31] C. Barnadas‐Solé , M. F. Zerón‐Rugerio , Á. Hernáez , J. Foncillas‐Corvinos , T. Cambras , and M. Izquierdo‐Pulido , “Late Bedtime Is Associated With Lower Weight Loss in Patients With Severe Obesity After Sleeve Gastrectomy,” International Journal of Obesity 45, no. 9 (2021): 1967–1975, 10.1038/s41366-021-00859-6. 34017047

[32] T. T. Hansen , M. F. Hjorth , K. Sandby , et al., “Predictors of Successful Weight Loss With Relative Maintenance of Fat‐Free Mass in Individuals With Overweight and Obesity on an 8‐Week Low‐Energy Diet,” British Journal of Nutrition 122, no. 04 (2019): 468–479, 10.1017/S0007114519001296. 31242952

[33] F. H. Mazri , Z. A. Manaf , S. Shahar , A. F. Mat Ludin , and N. A. Karim , “Improvement in Chrono‐Nutrition Is Associated With Robust Weight Loss Outcomes: An Extension of the Feasibility Study,” Chronobiology International 40, no. 3 (2023): 272–283, 10.1080/07420528.2023.2165092. 36803265

[34] M. S. Nielsen , H. Alsaoodi , M. F. Hjorth , and A. Sjödin , “Physical Activity, Sedentary Behavior, and Sleep Before and After Bariatric Surgery and Associations With Weight Loss Outcome,” Obesity Surgery 31, no. 1 (2021): 250–259, 10.1007/s11695-020-04908-3. 32803708

[35] S. Wen , Y. Ni , Y. Dai , et al., “Effects of a Calorie‐Restricted Dietary Intervention on Weight Loss and Gut Microbiota Diversity in Obese Patients With Sleep Deprivation,” Eating and Weight Disorders 28, no. 1 (2023): 80, 10.1007/s40519-023-01609-5. 37792102 PMC10550869

[36] C. E. Kline , E. R. Chasens , Z. Bizhanova , et al., “The Association Between Sleep Health and Weight Change During a 12‐Month Behavioral Weight Loss Intervention,” International Journal of Obesity 45, no. 3 (2021): 639–649, 10.1038/s41366-020-00728-8. 33414489 PMC7914147

[37] S. A. Creasy , D. M. Ostendorf , J. M. Blankenship , et al., “Effect of Sleep on Weight Loss and Adherence to Diet and Physical Activity Recommendations During an 18‐Month Behavioral Weight Loss Intervention,” International Journal of Obesity 46, no. 8 (2022): 1510–1517, 10.1038/s41366-022-01141-z. 35577898 PMC9850430

[38] M. Garaulet , J. M. Ordovás , and J. A. Madrid , “The Chronobiology, Etiology and Pathophysiology of Obesity,” International Journal of Obesity 34, no. 12 (2010): 1667–1683, 10.1038/ijo.2010.118. 20567242 PMC4428912

[39] M. L. Filiatrault , J. P. Chaput , V. Drapeau , and A. Tremblay , “Eating Behavior Traits and Sleep as Determinants of Weight Loss in Overweight and Obese Adults,” Nutrition & Diabetes 4, no. 10 (2014): e140, 10.1038/nutd.2014.37. 25329602 PMC4217000

[40] J. P. Chaput and A. Tremblay , “Sleeping Habits Predict the Magnitude of Fat Loss in Adults Exposed to Moderate Caloric Restriction,” Obesity Facts 5, no. 4 (2012): 561–566, 10.1159/000342054. 22854682

[41] R. Sawamoto , T. Nozaki , T. Furukawa , et al., “Higher Sleep Fragmentation Predicts a Lower Magnitude of Weight Loss in Overweight and Obese Women Participating in a Weight‐Loss Intervention,” Nutrition & Diabetes 4, no. 10 (2014): e144, 10.1038/nutd.2014.41. 25347608 PMC4217002

[42] A. C. Carvalho , L. P. Marot , L. A. Mattar , et al., “Associations of Subjective Sleep Patterns and Social Jet Lag With Weight Loss and Dietary Intake in Bariatric Surgery Patients: A 1‐Year Follow‐Up Study,” British Journal of Nutrition 133, no. 5 (2025): 711–720, 10.1017/S0007114525000352. 39991887

[43] T. N. Swanson , V. Bauman , and K. M. Ross , “Associations Between Chronotype, Weight Loss, and Adherence to Caloric Intake and Physical Activity Goals During a Behavioral Weight Loss Intervention,” Journal of Behavioral Medicine 48, no. 4 (2025): 722–729, 10.1007/s10865-025-00573-y. 40335844 PMC12606675

[44] N. S. Marshall , R. R. Grunstein , M. Peltonen , K. Stenlof , J. Hedner , and L. V. Sjostrom , “Changes in Sleep Duration and Changes in Weight in Obese Patients: The Swedish Obese Subjects Study,” Sleep and Biological Rhythms 8, no. 1 (2010): 63–71, 10.1111/j.1479-8425.2010.00431.x.

[45] A. Hinz , H. Glaesmer , E. Brähler , et al., “Sleep Quality in the General Population: Psychometric Properties of the Pittsburgh Sleep Quality Index, Derived From a German Community Sample of 9284 People,” Sleep Medicine 30 (2017): 57–63, 10.1016/j.sleep.2016.03.008. 28215264

[46] M. B. Raniti , J. M. Waloszek , O. Schwartz , N. B. Allen , and J. Trinder , “Factor Structure and Psychometric Properties of the Pittsburgh Sleep Quality Index in Community‐Based Adolescents,” Sleep 41, no. 6 (2018): zsy066, 10.1093/sleep/zsy066. 29608755

[47] M. W. A. Johns , “A New Method for Measuring Daytime Sleepiness: The Epworth Sleepiness Scale,” Sleep: Journal of Sleep Research & Sleep Medicine 14, no. 6 (1991): 540–545, 10.1093/sleep/14.6.540. 1798888

[48] A. P. Knowlden , M. Ottati , M. McCallum , and J. P. Allegrante , “The Relationship Between Sleep Quantity, Sleep Quality and Weight Loss in Adults: A Scoping Review,” Clinical Obesity 14, no. 2 (2024): e12634, 10.1111/cob.12634. 38140746 PMC10939867

[49] M. Goerke , U. Sobieray , A. Becke , E. Düzel , S. Cohrs , and N. G. Müller , “Successful Physical Exercise‐Induced Weight Loss Is Modulated by Habitual Sleep Duration in the Elderly: Results of a Pilot Study,” Journal of Neural Transmission 124, no. 1 (2017): 153–162, 10.1007/s00702-015-1460-y. 26403683

[50] E. Papatriantafyllou , D. Efthymiou , E. Zoumbaneas , C. A. Popescu , and E. Vassilopoulou , “Sleep Deprivation: Effects on Weight Loss and Weight Loss Maintenance,” Nutrients 14, no. 8 (2022): 8, 10.3390/nu14081549. PMC9031614 35458110

[51] C. Ding , L. L. Lim , L. Xu , and A. P. S. Kong , “Sleep and Obesity,” Journal of Obesity & Metabolic Syndrome 27, no. 1 (2018): 4–24, 10.7570/jomes.2018.27.1.4. 31089536 PMC6489488

[52] M. P. St‐Onge , “Sleep–Obesity Relation: Underlying Mechanisms and Consequences for Treatment,” Obesity Reviews 18, no. S1 (2017): 34–39, 10.1111/obr.12499. 28164452 PMC13098705

[53] N. R. W. Geiker , A. Astrup , M. F. Hjorth , A. Sjödin , L. Pijls , and C. R. Markus , “Does Stress Influence Sleep Patterns, Food Intake, Weight Gain, Abdominal Obesity and Weight Loss Interventions and Vice Versa?,” Obesity Reviews 19, no. 1 (2018): 81–97, 10.1111/obr.12603. 28849612

[54] H. S. Dashti and J. M. Ordovás , “Genetics of Sleep and Insights Into Its Relationship With Obesity,” Annual Review of Nutrition 41, no. 2021 (2021): 223–252, 10.1146/annurev-nutr-082018-124258. 34102077

[55] N. Esmaeili , L. Gell , T. Imler , et al., “The Relationship Between Obesity and Obstructive Sleep Apnea in Four Community‐Based Cohorts: An Individual Participant Data Meta‐Analysis of 12,860 Adults,” EClinicalMedicine 83 (2025): 103221, 10.1016/j.eclinm.2025.103221. 40330547 PMC12051718

[56] R. Tabrizi , P. Saneei , K. B. Lankarani , et al., “The Effects of Caffeine Intake on Weight Loss: A Systematic Review and Dos‐Response Meta‐Analysis of Randomized Controlled Trials,” Critical Reviews in Food Science and Nutrition 59, no. 16 (2019): 2688–2696, 10.1080/10408398.2018.1507996. 30335479

[57] I. Clark and H. P. Landolt , “Coffee, Caffeine, and Sleep: A Systematic Review of Epidemiological Studies and Randomized Controlled Trials,” Sleep Medicine Reviews 31 (2017): 70–78, 10.1016/j.smrv.2016.01.006. 26899133

[58] B. Nussbaumer‐Streit , I. Klerings , A. I. Dobrescu , et al., “Excluding Non‐English Publications From Evidence‐Syntheses Did Not Change Conclusions: A Meta‐Epidemiological Study,” Journal of Clinical Epidemiology 118 (2020): 42–54, 10.1016/j.jclinepi.2019.10.011. 31698064

[59] R. Rosenberg , M. J. Breit , K. C. Clark , et al., “Examining the Role of Sleep in Body Weight Regulation and Implications for Obesity Treatment: A Narrative Review,” Obesity Reviews 27, no. 2 (2026): e70017, 10.1111/obr.70017. 40922448 PMC13574319

