# Supplementary material for: Sleep and Its Impact on the Success of Weight Loss Interventions in Adults With Excess Weight: A Systematic Review
Source: Obes Rev. 2026 Jul 20;27(9):e70199. doi: 10.1111/obr.70199 (PMC13460920; doi:10.1111/obr.70199)
Supplement: Supplementary file 1 [file OBR-27-e70199-s001.pdf]

Sleep and its impact on the success of weight loss interventions in adults with excess weight: A systematic review

Lauren Duan[1], Jessica Berryhill[1], Nichole Dai[1], Gabrielle Sica[1], Jean-Philippe Chaput[1-3]

[1]School of Epidemiology and Public Health, Faculty of Medicine,
University of Ottawa, Ottawa, Ontario, Canada.

[2]Healthy Active Living and Obesity Research Group,
Children's Hospital of Eastern Ontario Research Institute, Ottawa, Ontario, Canada.

[3]Department of Pediatrics, Faculty of Medicine, University of Ottawa, Ottawa, Ontario, Canada.

Corresponding Author: Jean-Philippe Chaput, Healthy Active Living and Obesity Research Group, Children's Hospital of Eastern Ontario Research Institute, 401 Smyth Road, Ottawa, Ontario, Canada, K1H 8L1. Phone: +1 613 737 7600 ext. 3683.
Email: jpchaput@cheo.on.ca
ORCID number: 0000-0002-5607-5736

Supplementary Material. Finalized search strategy.

Lines 1-19 are MEDLINE (Ovid MEDLINE(R) ALL 1946 to February 20, 2024)
Lines 20-38 are Embase (Embase Classic + Embase 1947 to February 20, 2024)
Lines 39-48 are CENTRAL (EBM Reviews - Cochrane Central Register of Controlled Trials January 2024)

1 exp sleep/
2 (Sleep* adj3 (qualit* or quantit* or length* or time* or duration* or increas* or improv* or decreas* or reduction*)).ti,ab,kf.
3 (Sleep* adj3 (timing or variab* or fragmentation* or satisf* or regular*)).ti,ab,kf.
4 (Sleep* adj3 (habit* or behavio?r* or pattern* or efficien*)).ti,ab,kf.
5 1 or 2 or 3 or 4
6 exp overweight/
7 (obese or obesity or overweight or over weight or overeat* or over eat* or overfeed* or over feed* or corpulent or adiposity or excess weight).ti,ab,kf.
8 ((body mass index or bmi) adj3 (greater or high or elevated or excess)).ti,ab,kf.
9 6 or 7 or 8
10 weight loss/ or "weight-loss".ti,ab,kf.
11 ((lose or loss or lost or reduction or reduce* or decrease* or change*) adj3 (weight or bmi or body mass index or adiposity)).ti,ab,kf.
12 (prevent* adj3 (weight adj3 (gain* or increase*))).ti,ab,kf.
13 10 or 11 or 12
14 5 and 9 and
15 exp animals/ not humans.sh.
16 14 not 15
17 limit 16 to (english or french)
18 limit 17 to (case reports or comment or editorial or letter)
19 17 not 18
20 exp sleep/
21 (Sleep* adj3 (qualit* or quantit* or length* or time* or duration* or increas* or improv* or decreas* or reduction*)).ti,ab,kw.
22 (Sleep* adj3 (timing or variab* or fragmentation* or satisf* or regular*)).ti,ab,kw.
23 (Sleep* adj3 (habit* or behavio?r* or pattern* or efficien*)).ti,ab,kw.
24 20 or 21 or 22 or 23
25 exp obesity/
26 (obese or obesity or overweight or over weight or overeat* or over eat* or overfeed* or over feed* or corpulent or adiposity or excess weight).ti,ab,kw.
27 ((body mass index or bmi) adj3 (greater or high or elevated or excess)).ti,ab,kw.
28 25 or 26 or 27
29 body weight loss/ or "weight-loss".ti,ab,kw.

30 ((lose or loss or lost or reduction or reduce* or decrease* or change*) adj3 (weight or bmi or body mass index or adiposity)).ti,ab,kw.

31 (prevent* adj3 (weight adj3 (gain* or increase*))).ti,ab,kw.

32 29 or 30 or 31

33 24 and 28 and 32

34 limit 33 to ((english or french) and embase)

35 limit 34 to conference abstract

36 34 not 35

37 limit 36 to (editorial or letter)

38 36 not 37

39 sleep*.ti,ab,kw.

40 (obese or obesity or overweight or over weight or overeat* or over eat* or overfeed* or over feed* or corpulent or adiposity or excess weight).ti,ab,kw.

41 ((body mass index or bmi) adj3 (greater or high or elevated or excess)).ti,ab,kw.

42 40 or 41

43 "weight-loss".ti,ab,kw.

44 ((lose or loss or lost or reduction or reduce* or decrease* or change*) adj3 (weight or bmi or body mass index or adiposity)).ti,ab,kw.

45 (prevent* adj3 (weight adj3 (gain* or increase*))).ti,ab,kw.

46 43 or 44 or 45

47 39 and 42 and 46

48 limit 47 to (english or french)

49 19 use medall

50 38 use emczd

51 48 use cctr

52 or/49-51

53 remove duplicates from 52
